# The genetic basis of conspicuous coloration in the Guadeloupean anole: Evolution by sexual and ecological selection

**DOI:** 10.1002/ece3.10266

**Published:** 2023-07-09

**Authors:** Nicholas G. Crawford, Thomas J. McGreevy, Sean P. Mullen, Christopher J. Schneider

**Affiliations:** ^1^ Department of Biology Boston University Boston Massachusetts USA; ^2^ Department of Natural Resources Science University of Rhode Island Kingston Rhode Island USA

**Keywords:** adaptation, *Anolis marmoratus*, cluster of differentiation 36, functional genomics, high‐pressure liquid chromatography, melanophilin

## Abstract

Understanding how natural selection acts on the genome and contributes to the process of speciation is a primary aim of the study of evolution. Here we used natural variation in two subspecies of the Guadeloupean anole (*Anolis marmoratus* ssp.), from the island of Guadeloupe in the Lesser Antilles, to explore the genomic basis of adaptation and speciation in *Anolis* lizards. These subspecies inhabit distinct ecological environments and display marked differences in adult male color and pattern. We sequenced the complete genomes of 20 anoles, 10 from each subspecies, at 1.4× coverage. We used genome‐wide scans of population differentiation, allele frequency spectrum, and linkage disequilibrium to characterize the genomic architecture within and between the subspecies. While most of the genome was undifferentiated, we observed five large divergent regions. Within these regions we identified blocks, 5 kb pairs in length, enriched for fixed single nucleotide polymorphisms. These blocks encompass 97 genes, two of which are candidate pigmentation genes. One is melanophilin (*mlph*), which helps transport melanosomes within melanocytes. The other is a cluster of differentiation 36 (*cd36*), which regulates carotenoid pigment sequestration. We used high‐pressure liquid chromatography to confirm that carotenoid pigments are significantly more abundant in the conspicuous orange‐pigmented skin of male *A*. *m*. *marmoratus* suggesting that *cd36* may be regulating pigment deposition in this tissue. We identified for the first time a carotenoid gene that is a potential target of divergent sexual selection and may be contributing to the early stages of speciation in *Anolis* lizards.

## Graphical abstract

We used a functional genomics approach to identify genes involved in the stark phenotypic differences between two anole subspecies. We identified two genes potentially involved in pigmentation and used high‐pressure liquid chromatography analyses to confirm the differential expression of one of the genes. To our knowledge, we are the first study to identify the cluster of differentiation 36 gene as a potential target of natural selection.

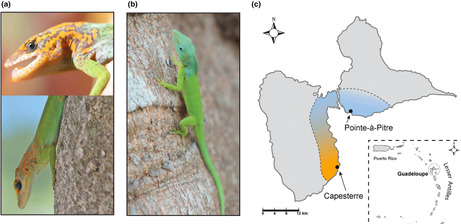

## 1 INTRODUCTION

Characterizing the genetic architecture of speciation is fundamental to understanding how new species evolve. While the mechanisms that drive speciation, such as natural selection, sexual selection, and genetic drift are well understood, there are few examples of how these processes act on the genome (Ellegren et al., 2012; Jones et al., 2012; Kronforst et al., 2013; Poelstra et al., 2014). Speciation research has traditionally focused on the genes that underlie reproductive isolation and not on those genes that contribute to the earliest stages of species divergence (Coyne & Orr, 2004; Endler, 1990; Moyle & Payseur, 2009; Orr et al., 2004; Presgraves, 2010). It can be argued that genes that initiate divergence are of greater interest to evolutionary biologists because reproductive isolation typically arises as a by‐product of selection, drift, or founder effects in isolated populations (Andersson, 1994; Kolbe et al., 2012; Ritchie, 2007; Shaw & Mullen, 2011). Thus, characterizing the genetic architecture of divergence allows for the identification of the genes and genomic loci vital to initiate the process of speciation.

Speciation can occur with varying degrees of connectivity between populations (Schluter, 2001) with new species arising in a continuum from sympatry, when the ranges of populations overlap entirely, to allopatry when populations are completely separated (Butlin et al., 2008). Here we focus on parapatric speciation, which occurs when populations diverge, but continue to overlap and interbreed for part of their range (Endler, 1977). Parapatric speciation is ubiquitous and is often driven by diversifying ecological selection along an environmental gradient (Doebeli & Dieckmann, 2003; Fu & Li, 1993). While divergent ecological selection is thought to play a central role in parapatric speciation (Kondrashov & Kondrashov, 1999; Schluter, 2009; Schneider, 1996), sexual selection also may contribute to divergence if the sexually selected trait is differentially detectable along the environmental gradient (Lande, 1982; Maan & Seehausen, 2011). Thus, populations in the earliest stages of parapatric speciation are expected to show divergence at genes and loci that contribute to phenotypes under ecological selection, sexual selection, or both processes.

Our goal was to characterize the genomic architecture of ecological diversification in two subspecies of *Anolis* lizards. Anoles are a model organism for the study of speciation, and represent one of the largest adaptive radiations of any terrestrial vertebrate (Losos, 2009). In the Caribbean, anoles have evolved similar body plans when species independently adapted to almost identical environments on different islands in what is considered a classic example of convergent evolution (Turner et al., 2005; Williams, 1972). Anoles also display a remarkable variety of colors and patterns both as adaptations for crypsis and for species and mate recognition. Most species of anoles possess a brightly colored extensible throat fan called a dewlap, which is used in inter‐ and intraspecific communication (Nicholson et al., 2007; Ord & Martins, 2006; Vanhooydonck et al., 2009). The differences in dewlap color and or pattern plays an important role restricting gene flow between species that is habitat dependent (Ng et al., 2017). However, in the early stages of speciation, anoles typically diverge first in male dorsal color and pattern (Losos, 2009), but see Stapley et al. (2011). This suggests that variation in dorsal coloration might arise as a result of natural selection, as well as female mate choice, although this has yet to be demonstrated. Dorsal coloration and pattern is shaped by both ecological and sexual selection as a consequence of local adaptation to spatially varying environments (Medina et al., 2017; Muñoz et al., 2013). Thus, divergence in dorsal coloration may initiate ecological divergence and be an important driver in speciation and adaptive radiation in anoles.

The bright colors of lizards are known to consist of two types of pigments: carotenoids and pteridines (reviewed in Olsson et al., 2013). Both carotenoid and pteridine pigments have been identified in the red and orange dewlaps of anoles (Macedonia et al., 2000). Although they produce similar colors, carotenoid pigments are sequestered from dietary sources (Olson & Owens, 1998) while pteridine pigments are synthesized endogenously from purines (i.e., guanosine‐5′‐triphosphate; Ziegler et al., 2000). Carotenoid pigments are of particular interest because they often color the bright integument of vertebrates (Goodwin, 1984), play important roles in immune function (Saks et al., 2003), and have antioxidant properties (Stahl & Sies, 2003). Thus, carotenoids are potentially honest indicators of quality if they color a sexually selected trait (Endler, 1980, 1983; Kodric‐Brown, 1985; Olson & Owens, 1998). However, carotenoid research on anole dewlap color has found no influence of food access or carotenoid supplementation (Steffen et al., 2010), but the role of carotenoids on anole dorsal color is unknown.

Although 11 candidate genes have been identified as potentially associated with carotenoid pigmentation (Walsh et al., 2012), the pathway that regulates carotenoid sequestration and deposition in vertebrates remains largely uncharacterized. Conversely, the genetic basis of pteridine pigment synthesis has been characterized in zebrafish and fruit flies (Dupont, 1958; Forrest & Mitchell, 1954), and is regulated by at least seven genes (Braasch et al., 2007; Kim et al., 2013; Ziegler et al., 2000). In anoles, De Mello et al. (2021) recently identified 13 candidate genes involved in anole dewlap color and color pattern. However, none of the genes in either pathway have been investigated in anole dorsal coloration. Because conspicuous coloration is one of the first traits to diverge in anoles, it is likely that either carotenoid or pteridine genes play a role in the speciation of anoles.

On the island of Guadeloupe in the Lesser Antilles, the Leopard or Guadeloupean anole (*Anolis marmoratus*) represents a unique example of an anole species in the early stages of parapatric speciation (Lazell, 1963, 1972; Muñoz et al., 2013; Schneider, 1996). *Anolis mamoratus* subspecies are primarily defined by differences in male color and pattern and are found in a variety of habitats ranging from wet cloud forest to arid scrub forest (Lazell, 1963). The two main islands are inhabited by six subspecies: five on Basse‐Terre and two on Grande‐Terre with one subspecies found on both islands (Lazell, 1963). Pairwise comparisons of mitochondrial DNA reveal very limited geographic structure between the subspecies (Schneider, 1996), and microsatellite loci genotyped in 11 populations on Grande‐Terre reveal no evidence of isolation by distance (Muñoz et al., 2013). A population genomic analysis also found high rates of gene flow among populations on both main islands (T. J. McGreevy, N. G. Crawford, P. Legreneur, & C. J. Schneider, unpublished data). This lack of genetic differentiation, combined with substantial phenotypic differences in male coloration, provides a unique opportunity to characterize the genetic architecture of phenotypic divergence. Importantly, because visual signals are key components of species recognition in anoles (Losos, 2009; Macedonia et al., 2013), our analysis is relevant to understanding the early stages of speciation in anoles.

**FIGURE 1:**
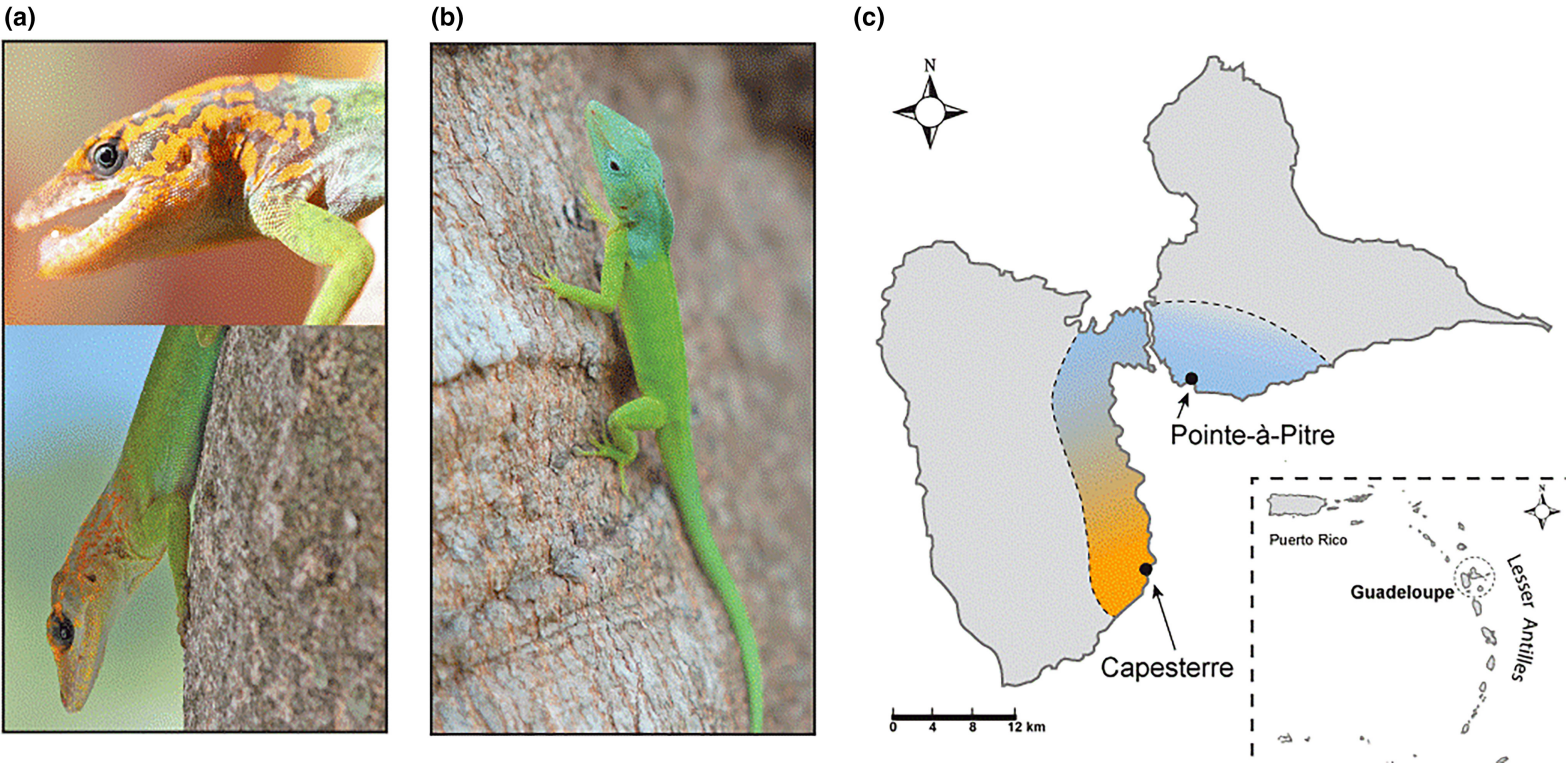
Representative photos of adult male *Anolis marmoratus*: (a) *Anolis m*. *marmoratus* from Capesterre and (b) *Anolis m*. *speciosus* from Pointe‐à‐Pitre. (c) depicts the approximate ranges of each subspecies with the color gradient representing the approximate transition between their dorsolateral head colors, the two collecting localities, and the clinal variation between them. The insert displays the location of the island of Guadeloupe relative to other islands in the Lesser Antilles.

Here we focus on two subspecies of *A*. *marmoratus*: *A*. *m*. *speciosus* and *A*. *m*. *marmoratus* that show a dramatic difference in coloration (Figure 1). *Anolis m*. *speciosus* inhabits the northeastern corner of Basse‐Terre and its range extends eastward into southwestern Grande‐Terre. While a narrow channel and mangrove swamps on both sides separates the islands, it does not appear to represent a physical barrier to gene flow because populations of *A*. *m*. *speciosus* on either side are essentially panmictic with very high rates of gene flow (T. J. McGreevy, N. G. Crawford, P. Legreneur, & C. J. Schneider, unpublished data). *Anolis m*. *speciosus* is found in mesic woodlands, and males have a conspicuous blue wash on their heads that is conspicuous in the light environment characteristic of their habitat (Muñoz et al., 2013). The blue coloration is consistent with expectations for effective signaling colors in large gaps in forests which are enriched for blue wavelengths of light (Endler, 1993). In contrast, *Anolis m*. *marmoratus* inhabits the southeastern coast of Basse‐Terre, and is found in closed tropical rainforest inhabiting what would be considered small gap light environments (sensu Endler, 1993). Males have conspicuous orange marbling on their heads consistent with expectations for conspicuous visual signals in small gap light environments which are enriched in the red‐orange wavelengths (Endler, 1993). There is clinal variation in head color across the range of the two subspecies with populations in the central eastern lowlands of Basse‐Terre displaying mixed phenotypes. The amount of orange on the head increases southward from just north of the town of Goyave and reaches its peak in the vicinity of Capesterre. Inversely, the amount of blue increases (and orange decreases) as one moves northward from south of Goyave to northeastern Basse‐Terre and southeastern Grande‐Terre, where blue coloration peaks in the vicinity of Pointe‐a‐Pitre. The conspicuously different phenotypes that we sampled in this study represent the end points of a cline in head coloration which exists despite the levels of gene flow sufficient to homogenize most of the genome. Our working hypothesis is that the conspicuous differences in head coloration result from divergent sexual selection (both intra‐ and inter‐ sexual selection) for effective signals driven by differences in light environment.

Using measures of light environment, skin reflectance, high‐performance liquid chromatography (HPLC) of carotenoid pigments, double digest restriction‐site‐associated DNA sequencing (ddRADseq), and low‐coverage whole genome sequencing we address the following questions in regard to the early stages of speciation in anoles: (1) which phenotypic traits are under divergent selection; (2) what is the genomic basis of divergence in those traits; and, because divergence involves orange coloration, (3) are genes associated with pteridine or carotenoid pigmentation involved? We hypothesize that due to the high rates of gene flow between *A*. *m*. *speciosus* and *A*. *m*. *marmoratus*, there will be small islands of divergence that are associated with genes involved in phenotypic divergence. And, because coloration plays an important role in speciation in the adaptive radiation of Caribbean *Anolis*, our findings will be relevant for understanding color evolution across the radiation.

## 2 MATERIALS AND METHODS

### 2.1 Sample collection and phenotyping

Lizards were collected on Guadeloupe in 2001, 2009, and 2012 under permits from the Prefecture de Guadeloupe and import approval to the United States by the United States Fish and Wildlife Service. Tail tip tissue samples were stored in 100% ethanol. An additional 20 live lizards, 10 *A*. *m*. *marmoratus* from Capesterre and 10 *A*. *m*. *speciosus* from Pointe‐à‐Pitre, were collected in 2012 to obtain skin samples for HPLC analyses. Lizards were euthanized and skin tissue was excised from the most pigmented portion of the dorsolateral region above and behind the eye. Tissues were immediately frozen in liquid nitrogen and transferred to a −80°C freezer for long‐term storage.

In addition to the tissue samples, phenotypic measurements were taken on live animals and included snout–vent length and spectral measurements from the dorsolateral head, eye‐ring, dorsolateral body, and dewlap. Spectral reflectance was measured with an Ocean Optics USB 2000 field‐portable spectrometer fitted with an Ocean Optics R400 UV‐VIS reflectance probe attached to a DT‐1000 tungsten halogen/deuterium UV‐VIS light source (Ocean Optics, Inc.). A white barium sulfate standard was used to zero the instrument. Special care was taken to ensure spectral measurements were not taken when the animals were stressed and in a darkened phenotype color. Reflectance measures were converted to hue following Endler (1990).

### 2.2 Whole genome library preparation and sequencing

Genomic DNA was extracted from tail tissue samples collected in 2001 using a Qiagen™ DNAEasy Tissue kit. Twenty libraries, one for each sample, were generated using a Nextera DNA Sample Prep Kit following the manufacturer's instructions (Illumina). Each library was indexed with a unique pair of Illumina barcodes. Sample concentrations were measured with a Qubit® 2.0 Fluorometer using a dsDNA HS Assay kit. Samples were pooled at equal concentrations and sequenced on an Illumina HiSeq with 101 bp paired‐end sequencing chemistry.

### 2.3 Double digest restriction‐site‐associated sequencing

ddRADseq makers were designed following Peterson et al. (2012) using the protocol available at http://www.bit.ly/ddRAD with minor modifications (Supplemental M1). Briefly, 48 *A*. *marmoratus* ssp. samples were collected from seven sites on Basse‐Terre and Grande‐Terre in either 2001 or 2009 (Table S1). Each sample had their genomic DNA standardized to 500 ng and was simultaneously digested with MluCl and NlaIII at 4°C overnight. Each sample was uniquely barcoded with adapters that ranged in size from 34 to 53 nucleotides. Samples were pooled at equal concentrations and subsequently size selected between 182 and 222 bp with a Pippin Prep® electrophoresis system (Sage Science). Samples were single‐read sequenced on one lane of 50 bp Illumina HiSeq™ 2000 sequencing system.

### 2.4 Quality control, read alignment, and SNP/indel calling

Reads were aligned to the *A*. *carolinensis* reference genome (AnoCar2, ensembl version 67) with Stampy (1.0.18, r1526). Stampy can incorporate prior information about expected sequence divergence from the reference genome, so we set the “substitution rate” option to 0.13 and ran Stampy with the “‐‐sensitive” flag set.

The Broad Institute's Genome Analysis Toolkit (GATK version 2.2‐16, g9f648cb) was used to identify single nucleotide variants (SNVs) and short insertions and deletions (indels). Following GATK's best practices, duplicate reads were identified with “MarkDuplicates” with Picard command line tools (version 1.72). Then indels were identified using GATK's “RealignerTargetCreator” and reads were locally realigned using “IndelRealigner”. SNVs and short indels were called using “UnifiedGenotyper” with permissive setting (“minimum base quality scores” were set to 2). Variants were then recalibrated using high‐quality SNVs identified in ddRAD dataset (SNV quality >500). Because we were concerned that the ddRAD dataset did not contain enough indels to provide an accurate truth set (~ 3 indels/thousand SNVs), we simply discarded those indels whose quality score fell into the lowest quantile scores.

We used BreakDancerMax (version 1.1, 2/21/2011) to identify inversions, translocations, and larger indels (Chen et al., 2009). Because BreakDancerMax identification requires high coverage to accurately identify structural variant, we applied the algorithm only at the level of populations (~14× coverage). We also only analyzed reads with mapping quality scores of at least five (i.e., reasonably likely to be uniquely mapped).

To calculate $G_{\mathrm{ST}}^{\prime \prime }$ (Meirmans & Hedrick, 2011) and to identify fixed SNPs, variant call format (VCF) files were processed with custom python code (https://github.com/ngcrawford/pypgen). We used ANNOVAR (Wang et al., 2010) to annotate SNVs that intersect with genes as well as to identify synonymous and non‐synonymous SNVs. Weir and Cockerham's *F*_ST_ (Weir & Cockerham, 1984) and Tajima's *D* (Tajima, 1989) for each subspecies was calculated with vcftools (Danecek et al., 2011). $G_{\mathrm{ST}}^{\prime \prime }$ and Tajima's *D* were calculated in 5 kilobase pair (kbp) nonoverlapping blocks, and only SNVs for which there were five samples per population were used in these calculations. We choose 5 kbp as our block size because this was the minimum block size where each block contained, on average, enough SNVs (*x* = 109.12 ± 52.00 stdev) to calculate summary statistics without sacrificing precision.

To measure the extent of linkage disequilibrium along the genome, we used Beagle (version 3.3.2) to phase our genotypes (Browning & Browning, 2007). We then used VCF tools to calculate correlation coefficients between all SNVs in 25 kbp nonoverlapping blocks. Blocks smaller than 25 kbp did not contain enough variants to accurately fit decay curves. Because *r* ^2^ is sensitive to rare alleles (Remington et al., 2001), we only included SNVs where the minor allele frequency was ≥0.2 and where the proportion of missing data was less than 20%. To each window we fitted a decay curve (Hill & Weir, 1988; Weir & Hill, 1986) and measured the fitted *r* ^2^ at the midpoint of the window (Alhaddad et al., 2013).

### 2.5 Carotenoid HPLC

Skin tissue was excised from the most pigmented portion of the dorsolateral region above and behind the eye. Tissues were immediately frozen in liquid nitrogen and transferred to a −80°C freezer for long‐term storage. All tissues were collected at the same time and stored in the dark.

All HPLC steps were performed at the Carotenoids and Health Laboratory at the Tufts School of Nutrition. Samples were extracted in a darkened room under red light to reduce photodegradation of pigments. After thawing, tissues were weighed on a Mettler Toledo AX26 Comparator high sensitivity balance. Each tissue was individually processed for HPLC analysis.

Each sample was homogenized in 5 mL chloroform/methanol (CHCl_3_:MeOH, 2:1 v/v) with an IKA® Ultra Turrax® T8 tissue homogenizer twice for 20 s. The Ultra Turrax® was rinsed with 5 mL CHCl_3_:MeOH and the two CHCl_3_:MeOH solutions were combined. To this volume, 1 mL of 0.85% saline and 100 μL of internal standard (echinenone diluted to absorb at 0.02) were added. The sample was then vortexed for 30 s.

To separate the CHCl_3_:MeOH mixture, the sample was centrifuged for 10 min at 800 *g* at 4°C. The lower CHCl_3_ layer was transferred to a fresh test tube and evaporated to dryness in a 40°C water bath. Additional pigments dissolved in the aqueous phase were extracted by adding 3 mL of hexane to the remaining aqueous layer, vortexed for 1 min, and centrifuged for 10 min at 800 *g*. The upper hexane layer was transferred to the dried sample and evaporated to dryness. The dried pigments were resuspended in 100 μL of 100% ethanol, vortexed for 1 min, sonicated for 30 s, and transferred to an autosampler vial for HPLC analysis.

### 2.6 HPLC analysis

Each sample was processed on a Waters HPLC instrument with a Waters 2996 Photodiode Array Detector, a Waters 2695 Separations Module, and a Waters 474 Scanning Fluorescence detector. A YMC(tm) Carotenoid column S‐3, 3.0 × 150 mm (# CT99S031503WT) was used to separate the pigments.

The running method used two solvents, A and B. Solvent A was composed of: 0.85% methanol, 0.12% methyl tert‐butyl ether (MBTE), 0.03% H_2_0, and 0.45 g/L ammonium acetate. Solvent B was composed of 0.08% methanol, 0.90% MBTE, 0.02% H_2_0, and 0.2 g/L ammonium acetate. Samples were run at a constant flow of 0.4 mL/min with A and B buffers. After the column was equilibrated, 20 μL of sample was injected. Then the following separation protocol was run: 21 min linear gradient from 100% solvent A to 45% solvent A, 1 min at 45% solvent A, 11 min linear gradient to 5% solvent A, 4 min at 5% solvent A, 2 min linear gradient to 100% solvent A, and 21 min at 100% solvent A. Peaks were identified using Empower 3 Software (Waters) and external calibration standards from Sigma–Aldrich.

## 3 RESULTS

### 3.1 Phenotypic measurements

To characterize differences in color and pattern between male *A*. *m*. *marmoratus* and *A*. *m*. *speciosus*, we measured the hue of the dewlap, dorsolateral head, skin around the eye (eye‐ring), dorsolateral body, and the tail. Hue relates measures of reflectance of the discrete colors in the visible spectrum (e.g., red, yellow, blue, and green; Endler, 1990). The only significant difference in pigmentation was in dorsolateral head coloration.

**FIGURE 2:**
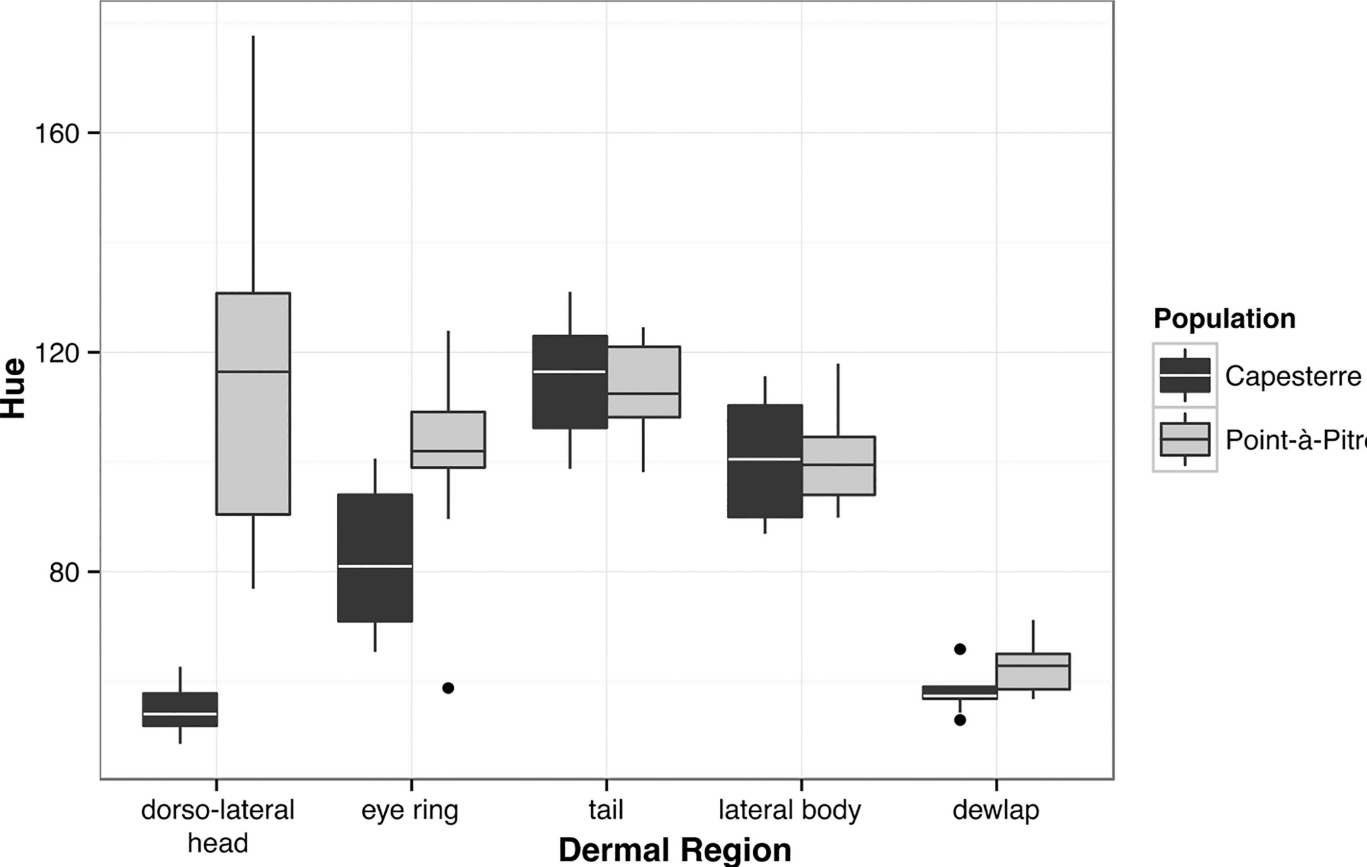
Mean hue measured from the head, eye‐ring, tail, lateral body, and dewlap of 20 male *Anolis marmoratus*: 10 *A*. *m*. *marmoratus* from Capesterre and 10 *A*. *m*. *speciosus* from Pointe‐à‐Pitre.

Color reflectance from ~32 millimeter skin patches on the dewlap, dorsolateral head, eye‐ring, dorsolateral body, and tail was measured from 10 individuals from each population (Figure 2). Reflectance was converted to hue following Endler and Mielke (2005; Table S2). A two factor ANOVA comparing hue of subspecies and skin patches (i.e., dewlap) showed significant differences in hue between populations [*F*(1) = 26.27, *p* < .001], skin patches [*F*(4) = 38.77, *p* < .001], and the interaction between pigments and patches [*F*(4) = 5.32, *p* < .001] (Figure 2; Table S3). A Tukey test identified 23 (*p* < .05) significant differences, but most were between different types of skin patches and did not explain differences between subspecies. Only the mean hue between dorsolateral head patches in *A*. *m*. *marmoratus* and *A*. *m*. *speciosus* was significantly different (*p* < .001).

**FIGURE 3:**
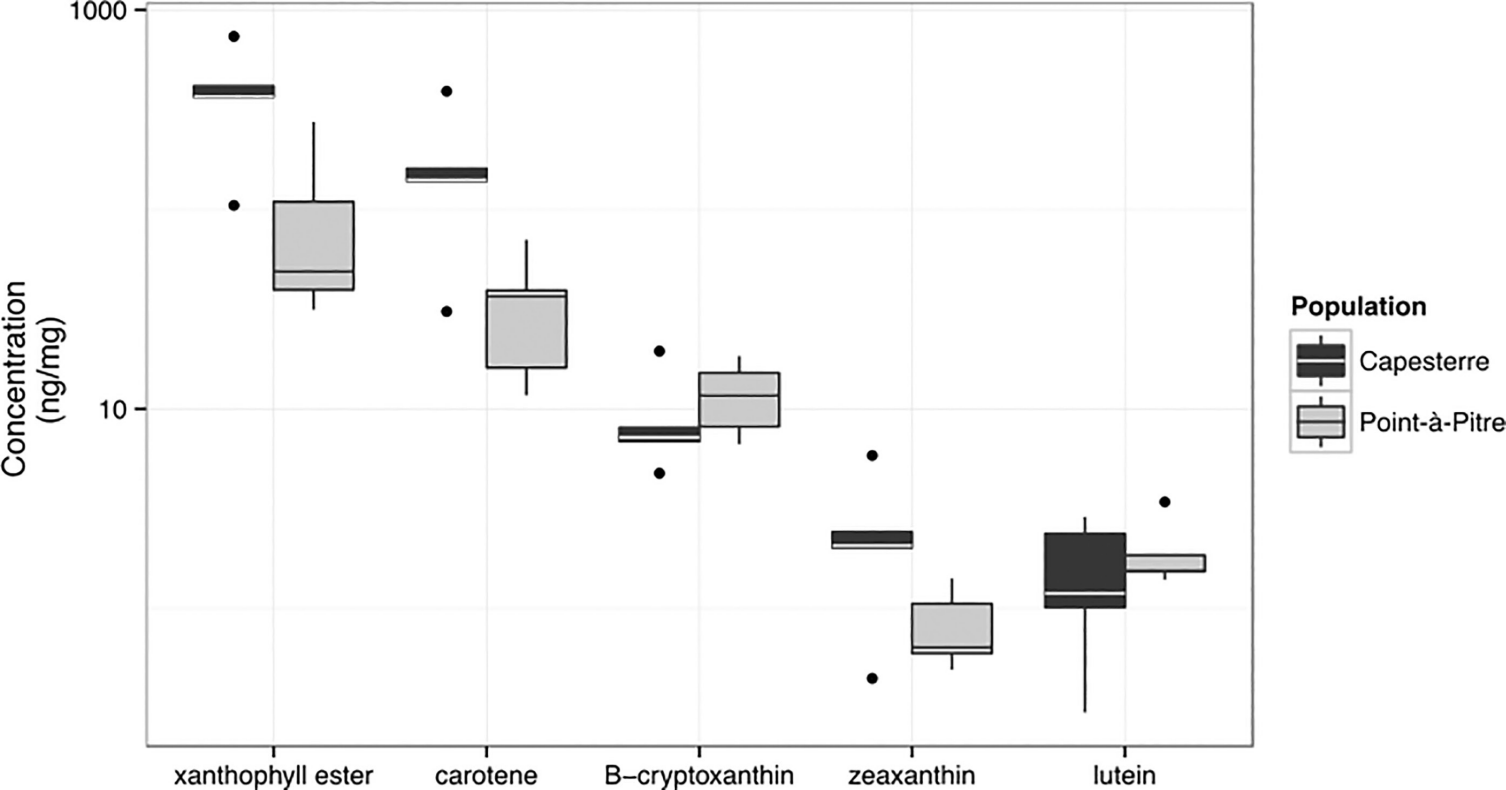
Mean carotenoid pigment content in the dorsolateral head from 10 *Anolis marmoratus*: five *A*. *m*. *marmoratus* from Capesterre and five *A*. *m*. *speciosus* from Pointe‐à‐Pitre. The *y*‐axis has been log transformed.

Because orange coloration may result from carotenoid pigments, we used HPLC to measure carotenoids in the head skin. We measured carotenoid pigments from five male *A*. *m*. *marmoratus* and five male *A*. *m*. *speciosus* (Figure 3; Table S4). Carotenoids were classified into five types: β‐cryptoxanthin, lutein, zeaxanthin, carotenes, and esterified xanthophylls (Figure S1). A two factor ANOVA comparing concentration of carotenoid pigments showed significant differences in concentration of carotenoid pigments between subspecies [*F*(1) = 11.895, *p* = .00134], pigments [*F*(4) = 14.580, *p* < .001], and the interaction between pigments and populations [*F*(4) = 5.521, *p* = .00123; Table S5]. A Tukey test identified nine significant differences (*p* < .05), but most were between different types of carotenoids and not biologically relevant. Only the mean concentration of esterified xanthophyll pigments between *A*. *m*. *marmoratus* and *A*. *m*. *speciosus* was significant (*p* < .001).

### 3.2 Sequencing and genomic variation

We sequenced 20 samples, 10 from *A*. *m*. *marmoratus* and 10 from *A*. *m*. *speciosus*, in two lanes of 101 bp paired‐end Illumina reads (Table S6). The complete dataset comprised ~628 million reads of which 93% aligned to the *A*. *carolinensis* genome (version 2; Alföldi et al., 2011). Mean coverage per sample was 1.4x ± 0.35 stdev. Of the 34 million SNVs that passed variant recalibration, only 7.6 million were variable within and among the two subspecies. The remaining 26.4 million are SNVs due to divergence from the reference genome and were not informative. We also identified 90,382 indels less than 11 bp in length and 56,314 larger structural variants of which there were 286 interchromosomal translocations, 18 deletions, 32 inversions, and 55,978 intrachromosomal translocations.

The ddRADseq produced 17,191,297 reads with 7,673,294 mapping to the *A*. *carolinensis* genome (Table S7). On average, 159,860 ± 72,763 (stdev) reads per sample mapped or 45% ± 3% (stdev).

### 3.3 Summary statistics

We used $G_{\mathrm{ST}}^{\prime \prime }$, an *F*‐statistic similar to *F*_ST_, to measure the degree of genetic differentiation between subspecies (Table 1). Unlike *F*_ST_, $G_{\mathrm{ST}}^{\prime \prime }$ accounts for multi‐allelic sites, small sample sizes, and a small number of sampled populations (Meirmans & Hedrick, 2011). We measured $G_{\mathrm{ST}}^{\prime \prime }$ across the genome in 5 kbp blocks (334,498 in total). Globally, mean $G_{\mathrm{ST}}^{\prime \prime }$ does not vary among chromosomes ($\bar{x}$ = 0.166 ± 0.106 stdev) with the exception of microchromosome LGb ($\bar{x}$ = 0.257 ± 0.148 stdev), which had a value 1.5 times higher. We identified both the top 1% (*N* = 3345, $\bar{x}$ = 0.5399) and 5% of nonoverlapping blocks (*N* = 16,725, $\bar{x}$ = 0.3569). We defined outliers as blocks falling within the top 1% of $G_{\mathrm{ST}}^{\prime \prime }$ outliers.

**TABLE 1:** Means of genomic parameters measured from 5 kbp windows including *F*_ST_, $G_{\mathrm{ST}}^{\prime \prime }$, Tajima's *D* calculated in 5 kbp blocks.

Parameter	Genomic background	Top 5% outliers	Max outlier
*F*_ST_ (Weir)	0.0387	0.3324	1.0000
$G_{\mathrm{ST}}^{\prime \prime }$	0.2102	0.2636	1.0000
Tajima's *D* *A*. *m*. *marmoratus*	0.7784	0.3198	2.7966
Tajima's *D* *A*. *m*. *speciosus*	0.4549	0.2396	2.6813
Linkage disequilibrium (*r* ^2^)	0.0242	0.05922	1.0770

*Note*: *r* ^2^ was calculated in 25 kb blocks. *F*_ST_ was calculated following Weir and Cockerham (1984) using VCFtools (Danecek et al., 2011). $G_{\mathrm{ST}}^{\prime \prime }$ was calculated with custom python code. Tajima's *D* was calculated with VCFtools. *r* ^2^ was calculated from a combination of R and custom python code. Genomic background includes all blocks with positive *F*_ST_ values excluding the top 5% of outliers.

We also calculated Tajima's *D*, a statistic which summarizes the allele frequency spectrum (Tajima, 1989). Tajima's *D* can be interpreted both in the context of demography and in the context of selection. Briefly, when Tajima's *D* is positive this indicates either a decrease in population size or balancing selection. Conversely, when Tajima's *D* is negative, this indicates either an increase in population size or purifying selection. However, low‐coverage sequencing is biased toward identifying homozygotes, which inflates estimates of Tajima's *D* (Korneliussen et al., 2013).

When viewed in the context of the entire genome there is a significant difference in the global means of Tajima's *D* between *A*. *m*. *marmoratus* ($\bar{x}$ = 0.770 ± 0.636 stdev) and *A*. *m*. *speciosus* ($\bar{x}$ = 0.462 ± 0.626 stdev; *t*‐test; *p* ≪ .001). Similar to $G_{\mathrm{ST}}^{\prime \prime }$, Tajima's *D* is significantly higher in both populations on microchromosome LGb than the genome‐wide average. In contrast, Tajima's *D* is significantly lower than the genome‐wide average in the top 1% of $G_{\mathrm{ST}}^{\prime \prime }$ outliers in both *A*. *m*. *marmoratus* (*t*‐test; *p* ≪ .001) and *A*. *m*. *speciosus* (*t*‐test; *p* = .03201).

**FIGURE 4:**
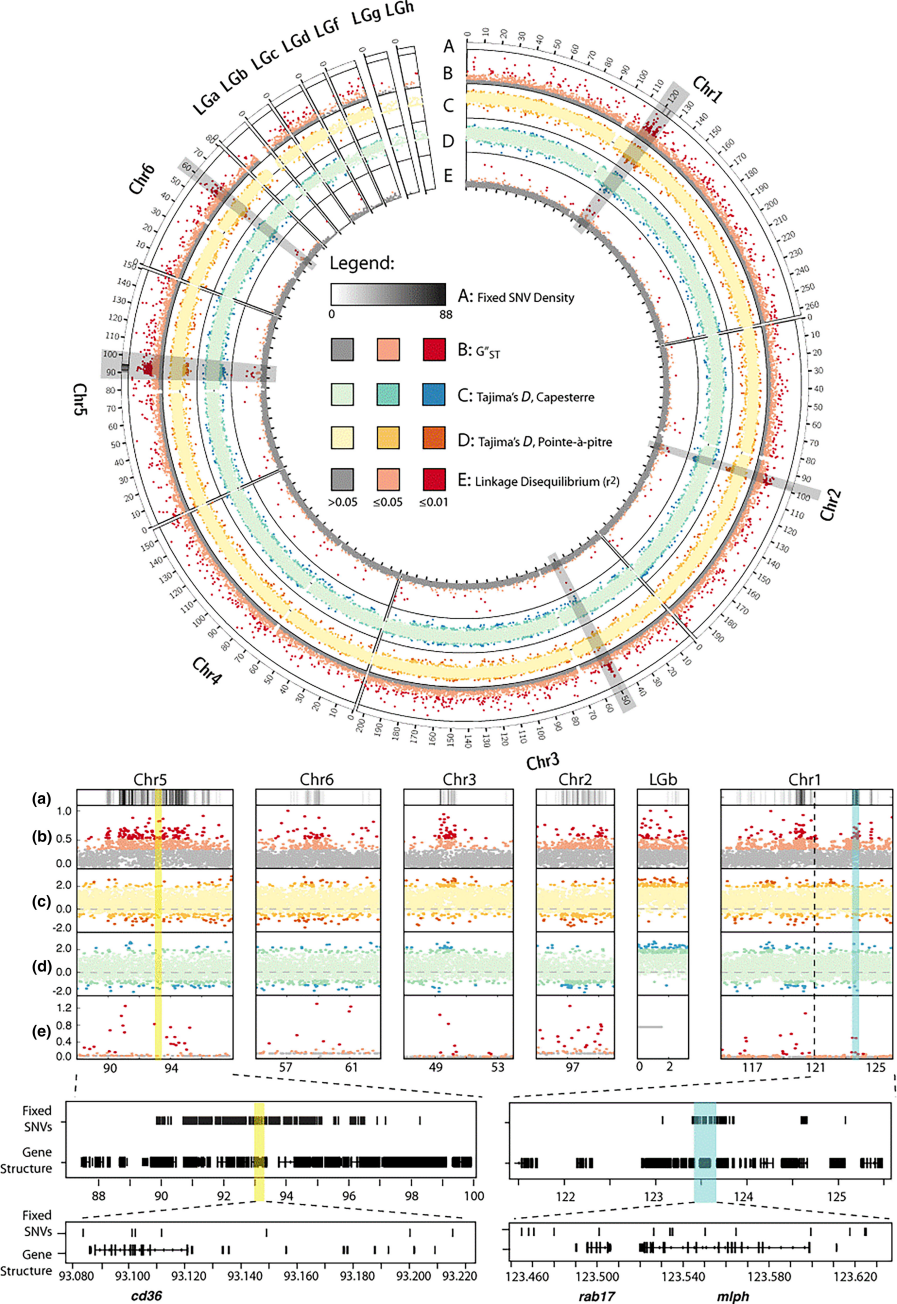
The upper circos plot displays fixed SNPs, $G_{\mathrm{ST}}^{\prime \prime }$, Tajima's *D*, and linkage disequilibrium (*r* ^2^). Tick marks are in units of megabases. Note that the scale of the microchromosomes has been increased for clarity. Regions of increased differentiation (blocks falling within the top 1% of $G_{\mathrm{ST}}^{\prime \prime }$ outliers) are highlighted with gray boxes. The lower panels show increasing degrees of magnification of the divergent regions. Note that the regions containing *cd36* and *mlph* are highlighted in yellow and blue, respectively.

**FIGURE 5:**
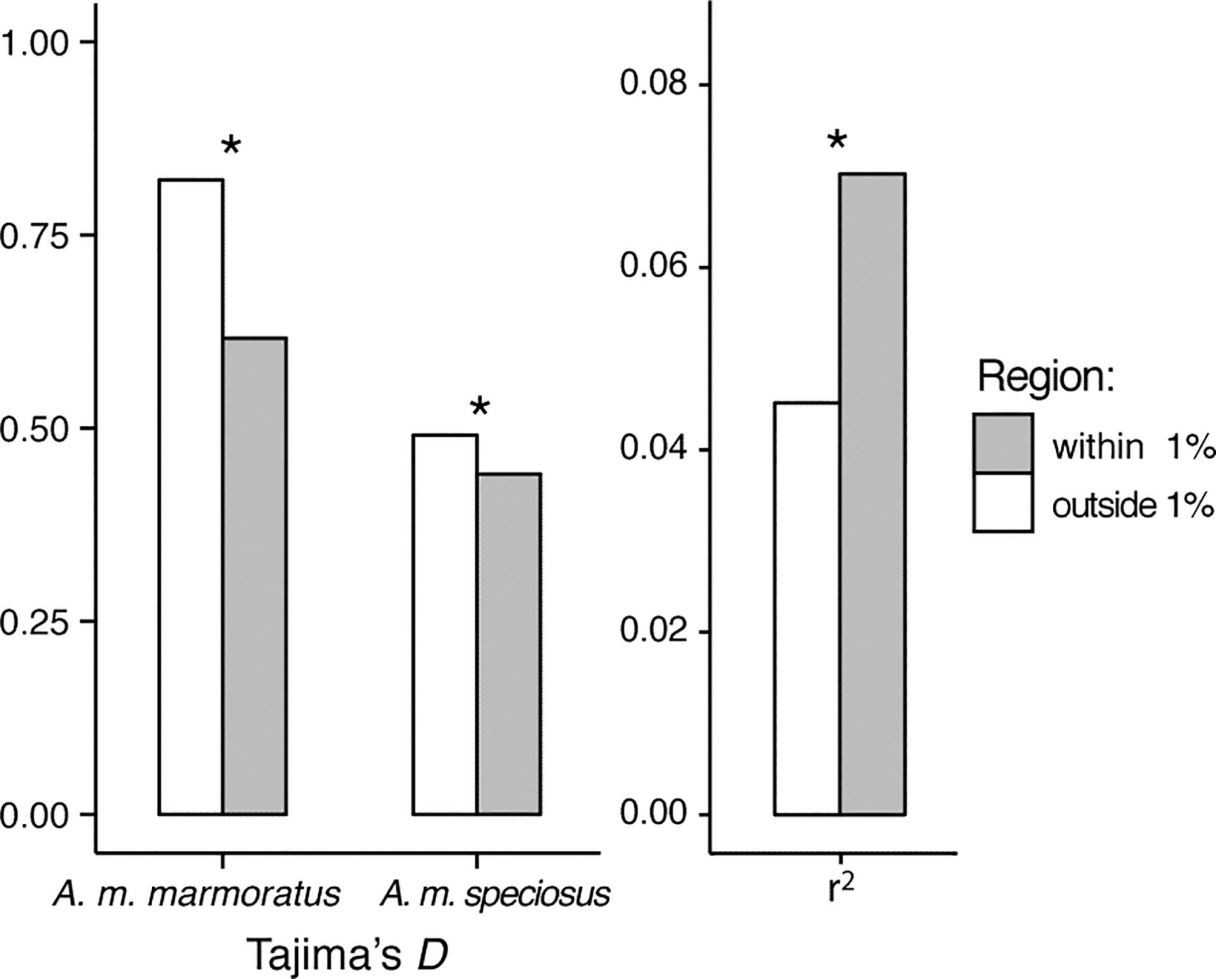
Bar plots and *r* ^2^ of Tajima's *D* in *A*. *m*. *marmoratus* and *A*. *m*. *speciosus* within and outside the 1% of *F*_ST_ outliers. Asterixis indicate significant difference in means as calculated by *t*‐test.

We identified five regions of increased divergence, when the top 1% of 5 kbp $G_{\mathrm{ST}}^{\prime \prime }$ blocks were physically clustered together on macrochromosomes 1, 2, 3, 5, and 6 (Figure 4). Within the clusters of outliers, Tajima's *D* was significantly reduced and LD was significantly increased relative to the rest of the genome at different alleles in Figure 5.

### 3.4 Diverging genes

Within the five divergent regions were 447 genes (*N* = 22,962; [Eckalbar et al., 2013]). These regions ranged from 1660 to 10,487 kbp, (Table 2), and contained genes potentially associated with pigmentation (*abca12*, *cd36*, *fox12*, *mlph*, *mocos*, *mreg*, *cd36*), spermatogenesis (*asun*, *rsbn1l*, *spag16*, *spats2l*), and thermoregulation (*trpm8*). When we examined the distribution of SNPs fixed between populations, we observed that they generally clustered at the center of the divergent regions. These regions intersect 97 genes of which only two, cluster of differentiation 36 (*cd36*) and melanophilin (*mlph*), are associated with pigmentation (Table S8). Eight SNVs fall within *mlph* introns and five within *cd36* introns.

**TABLE 2:** Positions of the five divergent regions and the means of $G_{\mathrm{ST}}^{\prime \prime }$, Tajima's *D*, and *r* ^2^ within these chromosome (Chrm) regions.

Chrm	Start	Stop	$G_{\mathrm{ST}}^{\prime \prime }$	Tajima's *D A. m. marmoratus*	Tajima's *D A*. *m*. *speciosus*	*r* ^2^
1	115,265,000	125,753,000	0.213424	0.717474	0.390429	.036700
2	98,089,000	99,749,000	0.269489	0.485895	0.036176	.049377
3	47,084,000	53,827,000	0.174782	0.79863	0.444996	.028969
5	88,289,000	97,998,000	0.254331	0.563555	0.237042	.034243
6	55,026,000	62,981,000	0.184175	0.629513	0.285627	.023194

## 4 DISCUSSION

Here we characterized dermal color differences in the adult males of two subspecies of *A*. *marmoratus*. We identified significant differences in hue and carotenoid content in the dorsolateral skin of the head. We show that genetic divergence occurs at only a few genomic regions comprising approximately 2% of their genome. In these regions, we identified two pigmentation genes: *mlph*, which is involved in melanosome transport, and *cd36*, which regulates carotenoid sequestration. These results suggest divergent selection on coloration, in this case likely sexual selection for effective visual signals, may initiate adaptive divergence in *A*. *marmoratus* populations and may be important in color divergence associated with speciation in anoles more broadly. However, direct tests of ecological and sexual selection are needed to determine the extent of their influence on divergence.

### 4.1 Phenotypic divergence

Because the head coloration is sex‐specific, conspicuous, and does not itself contribute to niche specialization, it is likely evolving primarily by sexual selection (Andersson, 1994; Ritchie, 2007). We hypothesize that local adaptation to light environment is a key to understanding divergence in color phenotypes. In particular, Endler's framework developed in his seminal 1993 paper, “The color of light in the forest and its implications” is relevant here. Endler (1993) shows that different light environments exist in the forest and suggests that visual signaling colors could be locally adapted to those light environments. We suspect that is what is happening in the anoles of Guadeloupe, which also has been seen with other anole species (e.g., *Anolis conspersus*; Macedonia, 2001). In general, *Anolis* populations in Guadeloupe are drab and cryptically colored if they live in open habitats where predation by visual predators may be important. This association of drab coloration and open habitats is consistent across the radiation of Caribbean *Anolis* (Losos, 2009), suggesting its adaptive importance. In contrast, Guadeloupean *Anolis* populations in mesic and wet forest habitats are generally conspicuous in their environment where even the green body color contrasts strongly with the background coloration (Muñoz et al., 2013). This pattern of general conspicuousness also was noted by Williams and Rand (1977) across species of *Anolis* more broadly. We hypothesize that the striking variation between the described subspecies *A*. *m*. *marmoratus* and *A*. *m*. *speciosus* represents local adaptation to different light environments at either end of a habitat and light environment cline between mesic forest and closed rainforest. Overall, the light environment, where the nominate subspecies *marmoratus* is found, is characteristic of light in the rainforest where it is dim overall, but small gaps generate a light environment enriched for red‐orange wavelengths (Endler, 1993). The habitat of the *speciosus* populations is, in contrast, more open and characterized by large gaps which are enriched for blue wavelengths (Endler, 1993). Therefore, we suspect that local adaptation of signaling colors to light environment is driving the divergence of these populations and that only a small portion of the genome is diverging—the remainder is homogenized by the high levels of overall gene flow among the populations. Because visual cues are a critical part of the *Anolis* species recognition system, divergence in color phenotypes and the associated genes has direct implications for understanding the early stages in the process of speciation in anoles more broadly.

### 4.2 Genomic architecture and identification of divergent regions

In both subspecies, genome‐wide genetic divergence, as measured by $G_{\mathrm{ST}}^{\prime \prime }$, and linkage disequilibrium are low. This suggests that gene flow is homogenizing much of their genome. High rates of gene flow and population migration also were found between *A*. *m*. *speciosus* and *A*. *m*. *inornatus* on the island of Grande‐Terre (Muñoz et al., 2013) and a recent analysis suggests that gene flow is high throughout the *Anolis marmoratus* complex on the two main islands (T. J. McGreevy, N. G. Crawford, P. Legreneur, & C. J. Schneider, unpublished data). Within subspecies, we observed slightly positive mean values of Tajima's *D*. Positive values of Tajima's *D* indicate population bottlenecks at a genome‐wide scale (Fu & Li, 1993). However, alternately, low‐coverage sequencing can produce an excess of homozygotes and singleton SNVs, thereby inflating measures of Tajima's *D* (Korneliussen et al., 2013). While we only used SNVs that passed variant recalibration, GATK's stringent allele calling may increase the number of homozygous genotypes because fewer reads are required to identify a homozygote. Therefore, we do not regard the positive Tajima's *D* values as biologically important.

Within the top 1% of outlier loci, we observed that Tajima's *D* was significantly lower than the genome‐wide average. Reduced Tajima's *D* is indicative of purifying selection which reduces genetic variation resulting in an excess of low frequency polymorphisms. We also observed increased linkage disequilibrium in these outlier loci. Increased linkage disequilibrium is expected in regions under purifying selection because decreased recombination increases the chance that selected alleles will remain in linkage disequilibrium. Together the regions of localized divergence combined with the reduced Tajima's *D* and increased linkage disequilibrium strongly suggest that selection is acting on the top 1% of divergent genomic regions.

We observed that these outliers appear to cluster into five divergent regions. Divergent regions are a common feature of speciation with gene flow and have been observed in butterflies, fish, and birds (Ellegren et al., 2012; Jones et al., 2012; Kronforst et al., 2013; Poelstra et al., 2014). In the literature these clusters have been variously described as “genomic islands of speciation” (Turner et al., 2005) or “genomic islands of divergence” (Ellegren et al., 2012; Jones et al., 2012; Kronforst et al., 2013; Nosil et al., 2009; Poelstra et al., 2014; Shaw & Mullen, 2011). These regions are thought to arise either by selection alone or due to inversions (Coyne & Orr, 2004; Cruickshank & Hahn, 2014; McGaugh & Noor, 2012; Moyle & Payseur, 2009; Nosil & Feder, 2012; Orr et al., 2004; Presgraves, 2010). We did not observe any evidence of inversions in our analysis and a parsimonious interpretation of our results is that selection has produced the divergent regions. However, structural variants, and inversions in particular, are difficult to identify with short‐read sequencing, so this conclusion will require further exploration (Lledó & Cáceres, 2013).

### 4.3 Genes in divergent regions

Because the divergent regions contained clusters of fixed SNVs, we identified 5 kbp blocks that contained at least one fixed SNV. This reduced the number of genes to 97. These blocks tended to cluster together in the center of the five divergent regions (Figure 4). This set of genes contained two potential pigmentation genes: *mlph* and *cd36*. Eight SNPs were within *mlph* introns and five within *cd36* introns. While the exons of both genes have coverage across all bases, in neither gene did a fixed SNP intersect a coding sequence. If either of these genes is contributing to the differences in pigmentation between the subspecies, this observation suggests that selection may be acting on regulation of gene expression. Additional research is needed to determine how coloration is being regulated at the gene level.

Neither *mlph* nor *cd36* are among known genes in the pteridine pigmentation pathway. *Mlph* regulates melanosome transport within melanocytes (Kuroda & Fukuda, 2004; Provance Jr. et al., 2002; Schluter, 2001; Wu et al., 2002). In humans and mice, mutations in *mlph* are characterized by reduced pigmentation in skin and hair (Matesic et al., 2001). However, phenotypic divergence between *A*. *m*. *marmoratus* and *A*. *m*. *speciosus* does not appear to involve differences in melanin pigmentation. Cluster of differentiation 36 is a transmembrane glycoprotein that helps direct the uptake of fatty acids (van Bennekum et al., 2005). In mice and silkworms, *cd36* appears to regulate the uptake of carotenoids (Doebeli & Dieckmann, 2003; Sakudoh et al., 2010, 2013; van Bennekum et al., 2005). Thus, *cd36* appears to be an important candidate locus to explain differences in carotenoid pigmentation.

### 4.4 HPLC

Because carotenoid pigments are known to be honest indicators of quality (Kondrashov & Kondrashov, 1999; Olson & Owens, 1998; Schluter, 2009), because *cd36* was a top hit in our genome scan, and because carotenoids usually produce orange or yellow pigmentation, we used HPLC to characterize the type and quantity of carotenoid pigments in the orange‐pigmented and non‐orange‐pigmented skin of the dorsolateral head of the two subspecies. We found that esterified‐xanthophyll esters were significantly more abundant in the conspicuous orange‐pigmented skin of *A*. *m*. *marmoratus*. While we did not measure pteridine pigments, the fact that we did not observe any genes associated with the pteridine biosynthetic pathway in our genome scan suggests that the pathway is not under selection in the two subspecies. Additional studies are necessary to determine whether *cd36* is differentially expressed in the epidermal tissue of *A*. *m*. *marmoratus*, but the HPLC analysis strongly suggests that differences in carotenoid abundance explain the differences in phenotype.

### 4.5 Sex chromosome divergence

When we investigated the divergence on macrochromosomes and microchromosomes, we observed that microchromosome LGb was significantly more divergent between the subspecies. This divergence is similar to the high divergence observed on male sex chromosomes in other pairs of incipient species such as *Ficedula* flycatchers (Ellegren et al., 2012). However, in *A*. *carolinensis* LGb is the female sex chromosome (Alföldi et al., 2011; Losos, 2009). Assuming that LGb also is the female sex chromosome in *A*. *marmoratus*, its divergence could have at least three explanations: (1) reduced effective population size of the female chromosome is increasing neutral divergence (Charlesworth et al., 1987; Williams, 1972); (2) selection on female genes in LGb is driving divergence (Charlesworth et al., 1987; Nicholson et al., 2007; Ord & Martins, 2006; Vanhooydonck et al., 2009); or (3) limited female dispersal which would result in increased divergence on the female chromosome.

Sex‐biased dispersal has been observed in other Lesser Antillean anoles (Johansson et al., 2008; Losos, 2009). Phylogenies generated from maternally inherited mitochondrial DNA in these subspecies form monophyletic groups (Schneider, 1996), which suggests that female dispersal may be limited. Population genetic theory suggests that male sex chromosomes should diverge even more rapidly because they do not recombine and are subject to more cell divisions (Graves, 2006; Maan & Seehausen, 2011). Thus, an additional possibility is that LGb may actually be the male sex chromosome in *A*. *m*. *marmoratus*.

## 5 CONCLUSION

The discovery that a gene (*cd36*) potentially involved in carotenoid sequestration is under divergent selection in *A*. *m*. *marmoratus* and *A*. *m*. *speciosus*, and that the conspicuously pigmented skin along the dorsolateral head of adult male *A*. *m*. *marmoratus* contains significantly more carotenoid pigments, suggests that *cd36* may be contributing to the differences in pigmentation between these two subspecies. This is particularly exciting because carotenoid pigments play important roles in sexually selected social signals and are considered to be honest indicators of quality (Olson & Owens, 1998; Olsson et al., 2013). Despite research on the genetic basis of carotenoid‐based phenotypes in reptiles and fish (García‐de Blas et al., 2013; Goodwin, 1984; Macedonia et al., 2000; Olson & Owens, 1998; Pike et al., 2010; Tripathi et al., 2009; Walsh et al., 2012; Ziegler et al., 2000), no carotenoid genes have been previously identified to be the targets of natural selection. Because *cd36* regulates the deposition of differently colored carotenoid pigments in mice and insects, our results suggest that it may be a key gene regulating the deposition of carotenoid pigments in animals. Gene expression and immunohistological analysis of *cd36* and *mlph* will help further characterize whether these genes are active in the pigmented epidermis of *A*. *m*. *marmoratus* and *A*. *m*. *speciosus*.

From a broader perspective, sexual selection and the incidence of sexual dichromatism have been shown to correlate with increased species diversity (Barraclough et al., 1995; Saks et al., 2003). *Anolis* lizards are remarkably diverse, with more than 400 described species and sympatric species almost always have different colored, conspicuous dewlaps (Losos, 2009; Nicholson et al., 2007; Williams & Rand, 1977). Within anoles, differences in coloration appear to evolve prior to the evolution of larger morphological changes, suggesting that divergence in pigmentation is an important component of speciation in anoles (Endler, 1980, 1983; Losos, 2009). The regions of clustered divergence observed in our study and the genes contained within them provide insight into the early stages of speciation within anoles. Future work in similar anoline systems such as the *A*. *distichus*, *brevirostris*, and *apletophallus* species complexes will help characterize whether the same regions and genes are contributing to early stages of speciation in anoles (Lambert et al., 2013; Ng & Glor, 2011; Olson & Owens, 1998; Stapley et al., 2011). Additional studies of expression and molecular evolution of *cd36* and *mlph* in a broad panel of vertebrates displaying carotenoid‐pigmented ornaments will help determine if these genes are important beyond the subspecies of *A*. *marmoratus*.

## AUTHOR CONTRIBUTIONS

**Nicholas G. Crawford:** Conceptualization (equal); data curation (lead); formal analysis (lead); investigation (equal); methodology (equal); project administration (equal); software (equal); validation (lead); visualization (lead); writing – original draft (lead); writing – review and editing (supporting). **Thomas J. McGreevy Jr.:** Data curation (supporting); funding acquisition (supporting); methodology (supporting); writing – original draft (supporting); writing – review and editing (lead). **Sean P. Mullen:** Conceptualization (supporting); data curation (supporting); formal analysis (supporting); investigation (supporting); methodology (supporting); visualization (supporting); writing – original draft (supporting); writing – review and editing (supporting). **Christopher J. Schneider:** Conceptualization (lead); data curation (equal); formal analysis (equal); funding acquisition (lead); investigation (lead); methodology (equal); project administration (lead); resources (lead); software (equal); supervision (lead); validation (equal); visualization (equal); writing – original draft (equal); writing – review and editing (equal).

## Supporting information


Appendix S1.

## Data Availability

Illumina sequence reads are available in NCBI's SRA database under BioProject accession numbers PRJNA990338 and PRJNA990481. Spectral reflectance measurements and HPLC table of summary statistics are available on datadryad (https://doi.org/10.5061/dryad.hdr7sqvp4).

## References

[ece310266-bib-0001] Alföldi, J. , di Palma, F. , Grabherr, M. , Williams, C. , Kong, L. , Mauceli, E. , Russell, P. , Lowe, C. B. , Glor, R. , Jaffe, J. D. , Ray, D. A. , Boissinot, S. , Shedlock, A. M. , Botka, C. , Castoe, T. A. , Colbourne, J. K. , Fujita, M. K. , Moreno, R. G. , ten Hallers, B. F. , … Linblad‐Toh, K. (2011). The genome of the green anole lizard and a comparative analysis with birds and mammals. Nature, 477, 587–591. 21881562 10.1038/nature10390 PMC3184186

[ece310266-bib-0002] Alhaddad, H. , Khan, R. , Grahn, R. A. , Gandolfi, B. , Mulikin, J. C. , Cole, S. A. , Gruffydd‐Jones, T. J. , Häggström, J. , Lohi, H. , Longeri, M. , & Lyons, L. A. (2013). Extent of linkage disequilibrium in the domestic cat, *Felis silvestris catus*, and its breeds. PLoS One, 8, e53537. 23308248 10.1371/journal.pone.0053537 PMC3538540

[ece310266-bib-0003] Andersson, M. (1994). Sexual selection. J. R. Krebs, & T. Clutton‐Brock (Eds.). Princeton University Press.

[ece310266-bib-0004] Barraclough, T. G. , Harvey, P. H. , & Nee, S. (1995). Sexual selection and taxonomic diversity in passerine birds. Proceedings of the Royal Society B: Biological Sciences, 259, 211–215.

[ece310266-bib-0005] Braasch, I. , Schartl, M. , & Volff, J.‐N. (2007). Evolution of pigment synthesis pathways by gene and genome duplication in fish. BMC Evolutionary Biology, 7, 74. 17498288 10.1186/1471-2148-7-74 PMC1890551

[ece310266-bib-0006] Browning, B. L. , & Browning, S. R. (2007). Efficient multilocus association testing for whole genome association studies using localized haplotype clustering. Genetic Epidemiology, 31, 365–375. 17326099 10.1002/gepi.20216

[ece310266-bib-0007] Butlin, R. K. , Galindo, J. , & Grahame, J. W. (2008). Sympatric, parapatric or allopatric: The most important way to classify speciation? Philosophical Transactions of the Royal Society, B: Biological Sciences, 363, 2997–3007. 10.1098/rstb.2008.0076 PMC2607313 18522915

[ece310266-bib-0008] Charlesworth, B. , Coyne, J. A. , & Barton, N. H. (1987). The relative rates of evolution of sex‐chromosomes and autosomes. The American Naturalist, 130, 113–146.

[ece310266-bib-0009] Chen, K. , Wallis, J. W. , McLellan, M. D. , Larson, D. E. , Kalicki, J. M. , Pohl, C. S. , McGrath, S. D. , Wendl, M. C. , Zhang, Q. , Locke, D. P. , Shi, X. , Fulton, R. S. , Ley, T. J. , Wilson, R. K. , Ding, L. , & Mardis, E. R. (2009). BreakDancer: An algorithm for high‐resolution mapping of genomic structural variation. Nature Methods, 6, 677–681. 19668202 10.1038/nmeth.1363 PMC3661775

[ece310266-bib-0010] Coyne, J. A. , & Orr, H. A. (2004). Speciation. Sinauer.

[ece310266-bib-0011] Cruickshank, T. E. , & Hahn, M. W. (2014). Reanalysis suggests that genomic islands of speciation are due to reduced diversity, not reduced gene flow. Molecular Ecology, 23, 3133–3157. 24845075 10.1111/mec.12796

[ece310266-bib-0012] Danecek, P. , Auton, A. , Abecasis, G. , Albers, C. A. , Banks, E. , DePristo, M. A. , Handsaker, R. E. , Lunter, G. , Marth, G. T. , Sherry, S. T. , McVean, G. , Durbin, R. , & 1000 Genomes Project Analysis Group . (2011). The variant call format and VCFtools. Bioinformatics, 27, 2156–2158. 21653522 10.1093/bioinformatics/btr330 PMC3137218

[ece310266-bib-0013] De Mello, P. L. H. , Hime, P. M. , & Glor, R. E. (2021). Transcriptomic analysis of skin color in anole lizards. Genome Biology and Evolution, 13, evab110. 33988681 10.1093/gbe/evab110 PMC8290120

[ece310266-bib-0014] Doebeli, M. , & Dieckmann, U. (2003). Speciation along environmental gradients. Nature, 421, 259–264. 12529641 10.1038/nature01274

[ece310266-bib-0015] Dupont, A. (1958). Pteridines in the scales of fishes. Naturwissenschaften, 45, 267–268.

[ece310266-bib-0016] Eckalbar, W. L. , Hutchins, E. D. , Markov, G. J. , Allen, A. N. , Corneveaux, J. J. , Linblad‐Toh, K. , Di Palma, F. , Alföldi, J. , Huentelman, M. J. , & Kusumi, K. (2013). Genome reannotation of the lizard *Anolis carolinensis* based on 14 adult and embryonic deep transcriptomes. BMC Genomics, 14, 49. 23343042 10.1186/1471-2164-14-49 PMC3561122

[ece310266-bib-0017] Ellegren, H. , Smeds, L. , Burri, R. , Olason, P. I. , Backström, N. , Kawakami, T. , Künstner, A. , Mäkinen, H. , Nadachowska‐Brzyska, K. , Qvarnström, A. , Uebbing, S. , & Wolf, J. B. W. (2012). The genomic landscape of species divergence in *Ficedula* flycatchers. Nature, 491, 756–760. 23103876 10.1038/nature11584

[ece310266-bib-0018] Endler, J. A. (1977). Geographic variation, speciation, and clines. Monographs in Population Biology, 10, 1–246. 409931

[ece310266-bib-0019] Endler, J. A. (1980). Natural‐selection on color patterns in *Poecilia‐Reticulata* . Evolution, 34, 76–91. 28563214 10.1111/j.1558-5646.1980.tb04790.x

[ece310266-bib-0020] Endler, J. A. (1983). Natural and sexual selection on color patterns in *poeciliid* fishes. Environmental Biology of Fishes, 9, 173–190.

[ece310266-bib-0021] Endler, J. A. (1990). On the measurement and classification of colour in studies of animal colour patterns. Biological Journal of the Linnean Society, 41, 315–352.

[ece310266-bib-0022] Endler, J. A. (1993). The color of light in forests and its implications. Ecological Monographs, 63, 1–27.

[ece310266-bib-0023] Endler, J. A. , & Mielke, P. (2005). Comparing entire colour patterns as birds see them. Biological Journal of the Linnean Society, 86, 405–431.

[ece310266-bib-0024] Forrest, H. S. , & Mitchell, H. K. (1954). Pteridines from *Drosophila*. I. Isolation of a yellow pigment1. Journal of the American Chemical Society, 76, 5656–5658.

[ece310266-bib-0025] Fu, Y. X. , & Li, W. H. (1993). Statistical tests of neutrality of mutations. Genetics, 133, 693–709. 8454210 10.1093/genetics/133.3.693 PMC1205353

[ece310266-bib-0026] García‐de Blas, E. , Mateo, R. , Viñuela, J. , Pérez‐Rodríguez, L. , & Alonso‐Alvarez, C. (2013). Free and esterified carotenoids in ornaments of an avian species: The relationship to color expression and sources of variability. Physiological and Biochemical Zoology, 86, 483–498. 23995480 10.1086/671812

[ece310266-bib-0027] Goodwin, T. W. (1984). The biochemistry of the carotenoids. Springer Netherlands.

[ece310266-bib-0028] Graves, J. A. M. (2006). Sex chromosome specialization and degeneration in mammals. Cell, 124, 901–914. 16530039 10.1016/j.cell.2006.02.024

[ece310266-bib-0029] Hill, W. G. , & Weir, B. S. (1988). Variances and covariances of squared linkage disequilibria in finite populations. Theoretical Population Biology, 33, 54–78. 3376052 10.1016/0040-5809(88)90004-4

[ece310266-bib-0030] Johansson, H. , Surget Groba, Y. , & Thorpe, R. S. (2008). Microsatellite data show evidence for male‐biased dispersal in the Caribbean lizard *Anolis roquet* . Molecular Ecology, 17, 4425–4432. 18803592 10.1111/j.1365-294X.2008.03923.x

[ece310266-bib-0031] Jones, F. C. , Grabherr, M. G. , Chan, Y. F. , Russell, P. , Mauceli, E. , Johnson, J. , Swofford, R. , Pirun, M. , Zody, M. C. , White, S. , Birney, E. , Searle, S. , Schmutz, J. , Grimwood, J. , Dickson, M. C. , Myers, R. M. , Miller, C. T. , Summers, B. R. , Knecht, A. K. , … Kingsley, D. M. (2012). The genomic basis of adaptive evolution in threespine sticklebacks. Nature, 484, 55–61. 22481358 10.1038/nature10944 PMC3322419

[ece310266-bib-0032] Kim, H. , Kim, K. , & Yim, J. (2013). Biosynthesis of drosopterins, the red eye pigments of *Drosophila melanogaster* . IUBMB Life, 65, 334–340. 23436441 10.1002/iub.1145

[ece310266-bib-0033] Kodric‐Brown, A. (1985). Female preference and sexual selection for male coloration in the guppy (*Poecilia reticulata*). Behavioral Ecology and Sociobiology, 17, 199–205.

[ece310266-bib-0034] Kolbe, J. J. , Leal, M. , Schoener, T. W. , Spiller, D. A. , & Losos, J. B. (2012). Founder effects persist despite adaptive differentiation: A field experiment with lizards. Science, 335, 1086–1089. 22300849 10.1126/science.1209566

[ece310266-bib-0035] Kondrashov, A. S. , & Kondrashov, F. A. (1999). Interactions among quantitative traits in the course of sympatric speciation. Nature, 400, 351–354. 10432111 10.1038/22514

[ece310266-bib-0036] Korneliussen, T. S. , Moltke, I. , Albrechtsen, A. , & Nielsen, R. (2013). Calculation of Tajima's D and other neutrality test statistics from low depth next‐generation sequencing data. BMC Bioinformatics, 14, 289. 24088262 10.1186/1471-2105-14-289 PMC4015034

[ece310266-bib-0037] Kronforst, M. R. , Hansen, M. , Crawford, N. G. , & Gallant, J. R. (2013). Hybridization reveals the evolving genomic architecture of speciation. Cell Reports, 5, 666–677. 24183670 10.1016/j.celrep.2013.09.042 PMC4388300

[ece310266-bib-0038] Kuroda, T. S. , & Fukuda, M. (2004). Rab27A‐binding protein Slp2‐a is required for peripheral melanosome distribution and elongated cell shape in melanocytes. Nature Cell Biology, 6, 1195–1203. 15543135 10.1038/ncb1197

[ece310266-bib-0039] Lambert, S. M. , Geneva, A. J. , Luke Mahler, D. , & Glor, R. E. (2013). Using genomic data to revisit an early example of reproductive character displacement in Haitian *Anolis* lizards. Molecular Ecology, 22, 3981–3995. 23551461 10.1111/mec.12292

[ece310266-bib-0040] Lande, R. (1982). Rapid origin of sexual isolation and character divergence in a cline. Evolution, 36, 213–223. 28563171 10.1111/j.1558-5646.1982.tb05034.x

[ece310266-bib-0041] Lazell, J. D. (1963). The anoles (Sauria, Iguanidae) of the Guadeloupeen archipelago. Bulletin of the Museum of Comparative Zoology, 131, 359–401.

[ece310266-bib-0042] Lazell, J. D. (1972). The anoles (Sauria, Iguanidae) of the Lesser Antilles. Bulletin of the Museum of Comparative Zoology, 143, 1–116.

[ece310266-bib-0043] Lledó, J. I. L. , & Cáceres, M. (2013). On the power and the systematic biases of the detection of chromosomal inversions by paired‐end genome sequencing. PLoS One, 8, e61292. 23637806 10.1371/journal.pone.0061292 PMC3634047

[ece310266-bib-0044] Losos, J. B. (2009). Lizards in an evolutionary tree: Ecology and adaptive radiation of anoles. University of California Press.

[ece310266-bib-0045] Maan, M. E. , & Seehausen, O. (2011). Ecology, sexual selection and speciation. Ecological Letters, 14, 591–602. 10.1111/j.1461-0248.2011.01606.x 21375683

[ece310266-bib-0046] Macedonia, J. M. (2001). Habitat light, colour variation, and ultraviolet reflectance in the grand Cayman anole, *Anolis conspersus* . Biological Journal of the Linnean Society, 73, 299–320.

[ece310266-bib-0047] Macedonia, J. M. , Clark, D. L. , Riley, R. G. , & Kemp, D. J. (2013). Species recognition of color and motion signals in *Anolis graham*: Evidence from responses to lizard robots. Behavioral Ecology, 24, 846–852.

[ece310266-bib-0048] Macedonia, J. M. , James, S. , Wittle, L. , & Clark, D. (2000). Skin pigments and coloration in the Jamaican radiation of *Anolis* lizards. Journal of Herpetology, 34, 99–109.

[ece310266-bib-0049] Matesic, L. E. , Yip, R. , Reuss, A. E. , Swing, D. A. , O'Sullivan, T. N. , Fletcher, C. F. , Copeland, N. G. , & Jenkins, N. A. (2001). Mutations in Mlph, encoding a member of the Rab effector family, cause the melanosome transport defects observed in leaden mice. Proceedings of the National Academy of Sciences of the United States of America, 98, 10238. 11504925 10.1073/pnas.181336698 PMC56945

[ece310266-bib-0050] McGaugh, S. E. , & Noor, M. A. F. (2012). Genomic impacts of chromosomal inversions in parapatric *drosophila* species. Philosophical Transactions: Biological Sciences, 367, 422–429. 22201171 10.1098/rstb.2011.0250 PMC3233717

[ece310266-bib-0094] Medina, I. , Losos, J. B. , & Mahler, D. L. (2017). Evolution of dorsal pattern variation in Greater Antillean Anolis lizards. Biological Journal of the Linnean Society, 120, 427–435.

[ece310266-bib-0051] Meirmans, P. , & Hedrick, P. (2011). Assessing population structure: FST and related measures. Molecular Ecology, 11, 5–8. 10.1111/j.1755-0998.2010.02927.x 21429096

[ece310266-bib-0052] Moyle, L. C. , & Payseur, B. A. (2009). Reproductive isolation grows on trees. Trends in Ecology & Evolution, 24, 591–598. 19717205 10.1016/j.tree.2009.05.010

[ece310266-bib-0053] Muñoz, M. M. , Crawford, N. G. , McGreevy, T. J., Jr. , Messana, N. J. , Tarvin, R. D. , Revell, L. J. , Zandvliet, R. M. , Hopwood, J. M. , Mock, E. , Schneider, A. L. , & Scheider, C. J. (2013). Divergence in coloration and ecological speciation in the *Anolis marmoratus* species complex. Molecular Ecology, 22, 2668–2682. 23611648 10.1111/mec.12295

[ece310266-bib-0093] Ng, J. , Geneva, A. J. , Noll, S. , & Glor, R. E. (2017). Signals and speciation: Anolis dewlap color as a reproductive barrier. Journal of Herpetology, 51, 437–447.

[ece310266-bib-0054] Ng, J. , & Glor, R. E. (2011). Genetic differentiation among populations of a Hispaniolan trunk anole that exhibit geographical variation in dewlap colour. Molecular Ecology, 20, 4302–4317. 21951523 10.1111/j.1365-294X.2011.05267.x

[ece310266-bib-0055] Nicholson, K. E. , Harmon, L. J. , & Losos, J. B. (2007). Evolution of *Anolis* lizard dewlap diversity. PLoS One, 2, e274. 17342208 10.1371/journal.pone.0000274 PMC1803026

[ece310266-bib-0056] Nosil, P. , & Feder, J. L. (2012). Genomic divergence during speciation: Causes and consequences. Philosophical Transactions of the Royal Society, B: Biological Sciences, 367, 332–342. 10.1098/rstb.2011.0263 PMC3233720 22201163

[ece310266-bib-0057] Nosil, P. , Funk, D. J. , & Ortiz‐Barrientos, D. (2009). Divergent selection and heterogeneous genomic divergence. Molecular Ecology, 18, 375–402. 19143936 10.1111/j.1365-294X.2008.03946.x

[ece310266-bib-0058] Olson, V. , & Owens, I. (1998). Costly sexual signals: Are carotenoids rare, risky or required? Trends in Ecology & Evolution, 13, 510–514. 21238418 10.1016/s0169-5347(98)01484-0

[ece310266-bib-0059] Olsson, M. , Stuart‐Fox, D. , & Ballen, C. (2013). Genetics and evolution of colour patterns in reptiles. Seminars in Cell & Developmental Biology, 24, 529–541. 23578866 10.1016/j.semcdb.2013.04.001

[ece310266-bib-0060] Ord, T. J. , & Martins, E. P. (2006). Tracing the origins of signal diversity in anole lizards: Phylogenetic approaches to inferring the evolution of complex behaviour. Animal Behavior, 71, 1411–1429.

[ece310266-bib-0061] Orr, H. A. , Masly, J. P. , & Presgraves, D. C. (2004). Speciation genes. Current Opinion in Genetics & Development, 14, 675–679. 15531163 10.1016/j.gde.2004.08.009

[ece310266-bib-0062] Peterson, B. K. , Weber, J. N. , Kay, E. H. , Fisher, H. S. , & Hoekstra, H. E. (2012). Double digest RADseq: An inexpensive method for de novo SNP discovery and genotyping in model and non‐model species. PLoS One, 7, e37135. 22675423 10.1371/journal.pone.0037135 PMC3365034

[ece310266-bib-0063] Pike, T. W. , Bjerkeng, B. , Blount, J. D. , Lindström, J. , & Metcalfe, N. B. (2010). How integument colour reflects its carotenoid content: A stickleback's perspective. Functional Ecology, 25, 297–304.

[ece310266-bib-0064] Poelstra, J. W. , Vijay, N. , Bossu, C. M. , Lantz, H. , Ryll, B. , Müller, I. , Baglione, V. , Unneberg, P. , Wikelski, M. , Grabherr, M. G. , & Wolf, J. B. W. (2014). The genomic landscape underlying phenotypic integrity in the face of gene flow in crows. Science, 344, 1410–1414. 24948738 10.1126/science.1253226

[ece310266-bib-0065] Presgraves, D. C. (2010). The molecular evolutionary basis of species formation. Nature Reviews Genetics, 11, 175–180. 10.1038/nrg2718 20051985

[ece310266-bib-0066] Provance, D. W., Jr. , James, T. L. , & Mercer, J. A. (2002). Melanophilin, the product of the leaden locus, is required for targeting of myosin‐Va to melanosomes. Traffic, 3, 124–132. 11929602 10.1034/j.1600-0854.2002.030205.x PMC1351229

[ece310266-bib-0067] Remington, D. L. , Thornsberry, J. M. , Matsuoka, Y. , Wilson, L. M. , Whitt, S. R. , Doebley, J. , Kresovich, S. , Goodman, M. M. , & Buckler, E. S. (2001). Structure of linkage disequilibrium and phenotypic associations in the maize genome. Proceedings of the National Academy of Sciences of the United States of America, 98, 11479–11484. 11562485 10.1073/pnas.201394398 PMC58755

[ece310266-bib-0068] Ritchie, M. G. (2007). Sexual selection and speciation. Annual Review of Ecology, Evolution, and Systematics, 38, 79–102.

[ece310266-bib-0070] Saks, L. , Ots, I. , & Hõrak, P. (2003). Carotenoid‐based plumage coloration of male greenfinches reflects health and immunocompetence. Oecologia, 134, 301–307. 12647136 10.1007/s00442-002-1125-z

[ece310266-bib-0071] Sakudoh, T. , Iizuka, T. , Narukawa, J. , Sezutsu, H. , Kobayashi, I. , Kuwazaki, S. , Banno, Y. , Kitamura, A. , Sugiyama, H. , Takada, N. , Fujimoto, H. , Kadano‐Okuda, K. , Mita, K. , Tamura, T. , Yamamoto, K. , & Tsuchida, K. (2010). A CD36‐related transmembrane protein is coordinated with an intracellular lipid‐binding protein in selective carotenoid transport for cocoon coloration. Journal of Biological Chemistry, 285, 7739–7751. 20053988 10.1074/jbc.M109.074435 PMC2844218

[ece310266-bib-0072] Sakudoh, T. , Kuwazaki, S. , Iizuka, T. , Narukawa, J. , Yamamoto, K. , Uchino, K. , Sezutsu, H. , Banno, Y. , & Tsuchida, K. (2013). CD36 homolog divergence is responsible for the selectivity of carotenoid species migration to the silk gland of the silkworm *Bombyx mori* . Journal of Lipid Research, 54, 482–495. 23160179 10.1194/jlr.M032771 PMC3588874

[ece310266-bib-0073] Schluter, D. (2001). Ecology and the origin of species. Trends in Ecology & Evolution, 16, 372–380. 11403870 10.1016/s0169-5347(01)02198-x

[ece310266-bib-0074] Schluter, D. (2009). Evidence for ecological speciation and its alternative. Science, 323, 737–741. 19197053 10.1126/science.1160006

[ece310266-bib-0075] Schneider, C. (1996). Distinguishing between primary and secondary intergradation among morphologically differentiated populations of *Anolis marmoratus* . Molecular Ecology, 5, 239–249. 8673270

[ece310266-bib-0076] Shaw, K. L. , & Mullen, S. P. (2011). Genes versus phenotypes in the study of speciation. Genetica, 139, 649–661. 21442403 10.1007/s10709-011-9562-4

[ece310266-bib-0077] Stahl, W. , & Sies, H. (2003). Antioxidant activity of carotenoids. Molecular Aspects of Medicine, 24, 345–351. 14585305 10.1016/s0098-2997(03)00030-x

[ece310266-bib-0078] Stapley, J. , Wordley, C. , & Slate, J. (2011). No evidence of genetic differentiation between anoles with different dewlap color patterns. Journal of Heredity, 102, 118–124. 20861275 10.1093/jhered/esq104

[ece310266-bib-0079] Steffen, J. E. , Hill, G. E. , & Guyer, C. (2010). Carotenoid access, nutritional stress, and the dewlap color of male brown anoles. Copeia, 2, 239–246.

[ece310266-bib-0080] Tajima, F. (1989). Statistical method for testing the neutral mutation hypothesis by DNA polymorphism. Genetics, 123, 585–595. 2513255 10.1093/genetics/123.3.585 PMC1203831

[ece310266-bib-0081] Tripathi, N. , Hoffmann, M. , Willing, E.‐M. , Lanz, C. , Weigel, D. , & Dreyer, C. (2009). Genetic linkage map of the guppy, *Poecilia reticulata*, and quantitative trait loci analysis of male size and colour variation. Proceedings of the Royal Society B: Biological Sciences, 276, 2195–2208. 10.1098/rspb.2008.1930 PMC2677598 19324769

[ece310266-bib-0082] Turner, T. L. , Hahn, M. W. , & Nuzhdin, S. V. (2005). Genomic islands of speciation in *Anopheles gambiae* . PLoS Biology, 3, e285. 16076241 10.1371/journal.pbio.0030285 PMC1182689

[ece310266-bib-0083] van Bennekum, A. , Werder, M. , Thuahnai, S. T. , Han, C.‐H. , Duong, P. , Williams, D. L. , Wettstein, P. , Schulthess, G. , Phillips, M. C. , & Hauser, H. (2005). Class B scavenger receptor‐mediated intestinal absorption of dietary beta‐carotene and cholesterol. Biochemistry, 44, 4517–4525. 15766282 10.1021/bi0484320

[ece310266-bib-0084] Vanhooydonck, B. , Herrel, A. , Meyers, J. J. , & Irschick, D. J. (2009). What determines dewlap diversity in *Anolis* lizards? An among‐Island comparison. Journal of Evolutionary Biology, 22, 293–305. 19196384 10.1111/j.1420-9101.2008.01643.x

[ece310266-bib-0085] Walsh, N. , Dale, J. , McGraw, K. J. , Pointer, M. A. , & Mundy, N. I. (2012). Candidate genes for carotenoid coloration in vertebrates and their expression profiles in the carotenoid‐containing plumage and bill of a wild bird. Proceedings of the Royal Society B: Biological Sciences, 279, 58–66. 10.1098/rspb.2011.0765 PMC3223654 21593031

[ece310266-bib-0086] Wang, K. , Li, M. , & Hakonarson, H. (2010). ANNOVAR: Functional annotation of genetic variants from high‐throughput sequencing data. Nucleic Acids Research, 38, e164. 20601685 10.1093/nar/gkq603 PMC2938201

[ece310266-bib-0087] Weir, B. S. , & Cockerham, C. C. (1984). Estimating F‐statistics for the analysis of population structure. Evolution, 38, 1358–1370. 28563791 10.1111/j.1558-5646.1984.tb05657.x

[ece310266-bib-0088] Weir, B. S. , & Hill, W. G. (1986). Nonuniform recombination within the human beta‐globin gene cluster. American Journal of Human Genetics, 38, 776–781. 3013006 PMC1684823

[ece310266-bib-0089] Williams, E. E. (1972). The origin of faunas. Evolution of lizard congeners in a complex Island fauna: A trial analysis. In T. Dobzhansky , M. K. Hecht , & W. C. Steere (Eds.), Evolutionary biology (pp. 47–89). Springer. 10.1007/978-1-4684-9063-3_3

[ece310266-bib-0090] Williams, E. E. , & Rand, A. S. (1977). Species recognition, dewlap function and faunal size. American Zoologist, 17, 261–270.

[ece310266-bib-0091] Wu, X. , Rao, K. , Zhang, H. , Wang, F. , Sellers, J. R. , Matesic, L. E. , Copeland, N. G. , Jenkins, N. A. , & Hammer, J. A. (2002). Identification of an organelle receptor for myosin‐Va. Nature Cell Biology, 4, 271–278. 11887186 10.1038/ncb760

[ece310266-bib-0092] Ziegler, I. , McDonald, T. , Hesslinger, C. , Pelletier, I. , & Boyle, P. (2000). Development of the pteridine pathway in the zebrafish, *Danio rerio* . Journal of Biological Chemistry, 275, 18926–18932. 10770954 10.1074/jbc.M910307199

