# Supplementary material for: The genetic basis of conspicuous coloration in the Guadeloupean anole: Evolution by sexual and ecological selection
Source: Ecol Evol. 2023 Jul 9;13(7):e10266. doi: 10.1002/ece3.10266 (PMC10330958; doi:10.1002/ece3.10266)
Supplement: Supplementary file 1 — Appendix S1. [file ECE3-13-e10266-s001.docx]

**Methods:**

**M1. RAD sequencing and analysis**

RAD libraries were created following Peterson et al., (2012). The full protocol is available here: <http://www.bit.ly/ddRAD>.

Extracted genomic DNA was quantified using the Invitrogen Qubit dsDNA HS Kit (ThermoFisher Scientific, Waltham, MA). Each sample was standardized to 500 ng of DNA and digested with MluCl (New England Biolabs, Ipswich, MA) and NlaIII (New England Biolabs, Ipswich, MA) according to manufacturer’s instructions. We used either Agencourt® AMPure XP magnetic beads (Beckman Coulter Genomics, Danvers, MA) or home-made Sera-Mag SpeedBeads (GE Healthcare, Pittsburgh, PA) magnetic beads (Rohland & Reich, 2012) for all purification steps. Digested and cleaned DNA samples were quantified using the Qubit High Sensitivity DNA Kit and all sets of samples were standardized to either 120 ng or 150 ng for the adapter ligation step. Samples were labeled with 12 adapters and purified using magnetic beads. Adapter ligated samples were quantified using the Qubit High Sensitivity DNA Kit and all samples were standardized to the same quantity. Sets of 12 samples with unique adapters were pooled and size selected for 202 base pairs (bp) with a range of 182 bp to 222 bp using 2% ethidium free cassettes on the Pippin Prep (Sage Science, Beverly, MA). Sets of four pools that each contained 12 samples were polymerase chain reaction (PCR) amplified to increase the quantity of each pool and incorporate a unique index. The KAPA HiFi™ Real-Time PCR Library Amplification Kit (Kapa Biosystems, Wilmington, MA) was used according to manufacturer’s instructions for 12 PCR cycles. Index labeled pools were purified and quantified using the Agilent High Sensitivity DNA kit (Agilent Technologies, Santa Clara, CA) according to manufacturer’s instructions. Equimolar amounts of each of the four pools were combined and purified. The final pool was quantified again using the Agilent High Sensitivity DNA kit and the library was submitted to the Northwest Laboratory Bauer Core Facility at Harvard University for single-end 50 bp read sequencing using an Illumina HiSeq™ 2000 sequencing system (San Diego, CA).

Table S1. Sample locality names for the areas where the double digest Restriction-site Associated DNA (ddRAD) analysis samples originated from. Location coordinates are in Universal Transverse Mercator (UTM) and the sample sizes (n) are given. Samples are listed from southeastern Basse Terre (Capesterre-Belle Eau) along the eastern coast to southwestern Grande Terre (Pointe-à-Pitre).

Locality Site X (UTM) Y (UTM) n Year Collected

Capesterre-Belle Eau CBE 653433.01 1775918.35 7 2001

Saint Marie SM 653644.15 1779149.65 7 2001

Goyave Go 652361.05 1783111.45 7 2009

Goyave North GoN 650920.74 1785634.36 7 2001

Plage de Viard PV 651212.63 1788144.07 7 2009

Plage au Petit Bourg PB 650744.12 1791022.9 7 2001

Pointe-à-Pitre PP 657088.28 1794398.88 6 2001

Table S2. Summarized spectral measurements from skin along the dorsolateral head. At least two reflectance measurements were taken from each lizard. To avoid pseudo-replication, we selected either the best measurement (the one with the smallest amount of external light contamination) or randomly choose one of the measurements. Summary statistics were calculated following (Endler & Mielke, 2005) and (Macedonia et al., 2003) . Columns are abbreviated as follows: ultraviolet (U) 325-399nm, blue (B) 400 - 474 nm, green (G) 475-549nm, yellow (Y) 550-624nm, red (R), 625-700, chroma (C), and hue (H).

| Sample ID | U | B | G | Y | R | C | H |
| --- | --- | --- | --- | --- | --- | --- | --- |
| CJS2289 | 0.09 | 0.12 | 0.18 | 0.31 | 0.30 | 0.24 | 60.42 |
| CJS2290 | 0.04 | 0.05 | 0.13 | 0.36 | 0.41 | 0.42 | 49.75 |
| CJS2291 | 0.11 | 0.08 | 0.15 | 0.35 | 0.31 | 0.32 | 59.31 |
| CJS2282 | 0.09 | 0.09 | 0.16 | 0.36 | 0.30 | 0.31 | 63.45 |
| CJS2283 | 0.10 | 0.12 | 0.17 | 0.31 | 0.31 | 0.24 | 55.02 |
| CJS2286 | 0.09 | 0.08 | 0.13 | 0.34 | 0.36 | 0.35 | 49.03 |
| CJS2288 | 0.12 | 0.12 | 0.17 | 0.30 | 0.29 | 0.21 | 56.16 |
| CJS2292 | 0.15 | 0.16 | 0.21 | 0.25 | 0.22 | 0.11 | 84.34 |
| CJS2293 | 0.14 | 0.15 | 0.20 | 0.28 | 0.23 | 0.14 | 77.94 |
| CJS2294 | 0.04 | 0.17 | 0.30 | 0.34 | 0.15 | 0.34 | 116.67 |
| CJS2295 | 0.12 | 0.17 | 0.26 | 0.29 | 0.16 | 0.20 | 118.51 |
| CJS2296 | 0.09 | 0.17 | 0.23 | 0.29 | 0.21 | 0.18 | 95.84 |
| CJS2297 | 0.05 | 0.17 | 0.30 | 0.34 | 0.15 | 0.34 | 115.61 |
| CJS2298 | 0.07 | 0.15 | 0.27 | 0.33 | 0.18 | 0.29 | 108.17 |
| CJS2299 | 0.20 | 0.22 | 0.26 | 0.21 | 0.11 | 0.17 | 156.77 |
| CJS2300 | 0.13 | 0.14 | 0.21 | 0.30 | 0.21 | 0.18 | 90.38 |

Table S3. The analysis of variance (ANOVA) reveals that all comparisons between subspecies and hue are significant (p < 0.05). The column names abbreviated as follows: degrees of freedom (df), sum of squares (sum sq), mean sum of squares (mean sq), F test statistic (F value), and p-value (Pr>(F)). The tukey HDS test identifies the comparison with the greatest difference in the observed means relevant difference is the comparison between dorso-lateral head hue. The column names are comparison, the difference in the observed means (diff), and the p-value after adjustment for the multiple comparisons (p adj). Subspecies and skin patch types are abbreviated as follows: *A. m. marmoratus* (A.m.mar) and *A. m. speciosus* (A. m. spe), dorso-lateral head (head_spot), lateral body (lat_body)

**ANOVA:**

|  | Df | Sum Sq | Mean Sq | F value | Pr(>F) |
| --- | --- | --- | --- | --- | --- |
| Subspecies | 1 | 5102 | 5102 | 26.27 | 1.98E-06 |
| Hue | 4 | 30120 | 7530 | 38.77 | < 2e-16 |
| Subspecies:Hue | 4 | 11901 | 2975 | 15.32 | 2.28E-09 |
| Residuals | 81 | 15732 | 194 |  |  |

**Tukey’s Honest Significant Difference (HSD) Test:**

|  | diff | p adj |
| --- | --- | --- |
| A.m.spe:head_spot-A.m.mar:head_spot | 60.44856381 | 0 |
| A.m.spe:eye_ring-A.m.mar:head_spot | 45.83482254 | 0 |
| A.m.mar:tail-A.m.mar:head_spot | 60.50228973 | 0 |
| A.m.spe:tail-A.m.mar:head_spot | 58.268159 | 0 |
| A.m.mar:dewlap-A.m.spe:head_spot | -57.34871538 | 0 |
| A.m.spe:dewlap-A.m.spe:head_spot | -52.65727026 | 0 |
| A.m.mar:dewlap-A.m.mar:tail | -57.4024413 | 0 |
| A.m.spe:dewlap-A.m.mar:tail | -52.71099618 | 0 |
| A.m.mar:dewlap-A.m.spe:tail | -55.16831058 | 0 |
| A.m.spe:dewlap-A.m.spe:tail | -50.47686546 | 0 |
| A.m.mar:lat_body-A.m.mar:head_spot | 46.42404979 | 0.0000001 |
| A.m.spe:lat_body-A.m.mar:head_spot | 45.48385748 | 0.0000001 |
| A.m.mar:dewlap-A.m.spe:eye_ring | -42.73497411 | 0.0000001 |
| A.m.mar:dewlap-A.m.mar:lat_body | -43.32420137 | 0.0000002 |
| A.m.mar:dewlap-A.m.spe:lat_body | -42.38400906 | 0.0000002 |
| A.m.spe:dewlap-A.m.spe:eye_ring | -38.04352899 | 0.0000015 |
| A.m.spe:dewlap-A.m.spe:lat_body | -37.69256394 | 0.0000019 |
| A.m.spe:dewlap-A.m.mar:lat_body | -38.63275625 | 0.000002 |
| A.m.mar:tail-A.m.mar:eye_ring | 32.7559203 | 0.0004908 |
| A.m.mar:eye_ring-A.m.spe:head_spot | -32.70219438 | 0.000505 |
| A.m.spe:tail-A.m.mar:eye_ring | 30.52178958 | 0.0011002 |
| A.m.mar:eye_ring-A.m.mar:head_spot | 27.74636943 | 0.0085197 |
| A.m.mar:dewlap-A.m.mar:eye_ring | -24.646521 | 0.0241971 |
| A.m.spe:dewlap-A.m.mar:eye_ring | -19.95507588 | 0.1209714 |
| A.m.mar:lat_body-A.m.mar:eye_ring | 18.67768036 | 0.208213 |
| A.m.spe:eye_ring-A.m.mar:eye_ring | 18.08845311 | 0.2193919 |
| A.m.spe:lat_body-A.m.mar:eye_ring | 17.73748805 | 0.2428869 |
| A.m.spe:lat_body-A.m.mar:tail | -15.01843225 | 0.3722583 |
| A.m.spe:lat_body-A.m.spe:head_spot | -14.96470633 | 0.3773895 |
| A.m.mar:tail-A.m.spe:eye_ring | 14.66746719 | 0.406354 |
| A.m.spe:eye_ring-A.m.spe:head_spot | -14.61374127 | 0.4116876 |
| A.m.mar:lat_body-A.m.mar:tail | -14.07823993 | 0.5036974 |
| A.m.mar:lat_body-A.m.spe:head_spot | -14.02451402 | 0.5092517 |
| A.m.spe:lat_body-A.m.spe:tail | -12.78430152 | 0.5664115 |
| A.m.spe:tail-A.m.spe:eye_ring | 12.43333647 | 0.6050117 |
| A.m.mar:lat_body-A.m.spe:tail | -11.84410921 | 0.7018539 |
| A.m.spe:dewlap-A.m.mar:head_spot | 7.79129355 | 0.9736153 |
| A.m.spe:dewlap-A.m.mar:dewlap | 4.69144512 | 0.999203 |
| A.m.mar:dewlap-A.m.mar:head_spot | 3.09984842 | 0.9999841 |
| A.m.spe:tail-A.m.mar:tail | -2.23413072 | 0.9999985 |
| A.m.spe:tail-A.m.spe:head_spot | -2.18040481 | 0.9999988 |
| A.m.mar:tail-A.m.spe:head_spot | 0.05372592 | 1 |
| A.m.mar:lat_body-A.m.spe:eye_ring | 0.58922726 | 1 |
| A.m.spe:lat_body-A.m.spe:eye_ring | -0.35096506 | 1 |
| A.m.spe:lat_body-A.m.mar:lat_body | -0.94019231 | 1 |

Table S4. Carotenoid pigments were measured on C_30_ HPLC column. Tissue was excised from the dorso-lateral head, frozen in liquid nitrogen, and stored in the dark at -80°C until extraction. Concentrations of β-cryptoxanthin, β-carotenes, lutein, xanthophyll esters, and zeaxanthin are in nanograms per microliter (ng/μL).

| Sample  ID | Tissue  Weight (mg) | β‑cryptoxanthin | β‑carotenes | lutein | xanthophyll esters | zeaxanthin |
| --- | --- | --- | --- | --- | --- | --- |
| CJS2282 | 1.76 | 4.77 | 160.37 | 2.37 | 365.62 | 2.02 |
| CJS2285 | 1.31 | 7.20 | 390.89 | 0.30 | 368.61 | 0.45 |
| CJS2286 | 1.295 | 8.06 | 30.77 | 1.19 | 104.73 | 2.42 |
| CJS2289 | 1.484 | 6.87 | 140.61 | 1.01 | 415.22 | 2.06 |
| CJS2290 | 0.534 | 19.49 | 138.77 | 2.88 | 735.33 | 5.85 |
| CJS2287 | 0.799 | 15.14 | 39.22 | 3.42 | 109.21 | 0.64 |
| CJS2292 | 0.941 | 11.67 | 16.07 | 1.53 | 31.45 | 1.41 |
| CJS2293 | 1.47 | 8.16 | 36.61 | 1.39 | 48.86 | 0.60 |
| CJS2295 | 0.78 | 18.40 | 70.52 | 1.85 | 273.84 | 1.06 |
| CJS2299 | 1.657 | 6.67 | 11.70 | 1.54 | 39.56 | 0.49 |

Figure S1. Exemplar Carotenoid HPLC chromatogram with labeled peaks. Chromatogram from C_30_ HPLC column of *A. m. marmoratus* (sample ID: CJS2282). Labeled peaks are lutein, zeaxanthin, β-cryptoxanthin, β-carotenes, xanthophyll esters, and an echinenone internal standard. AU is arbitrary units.


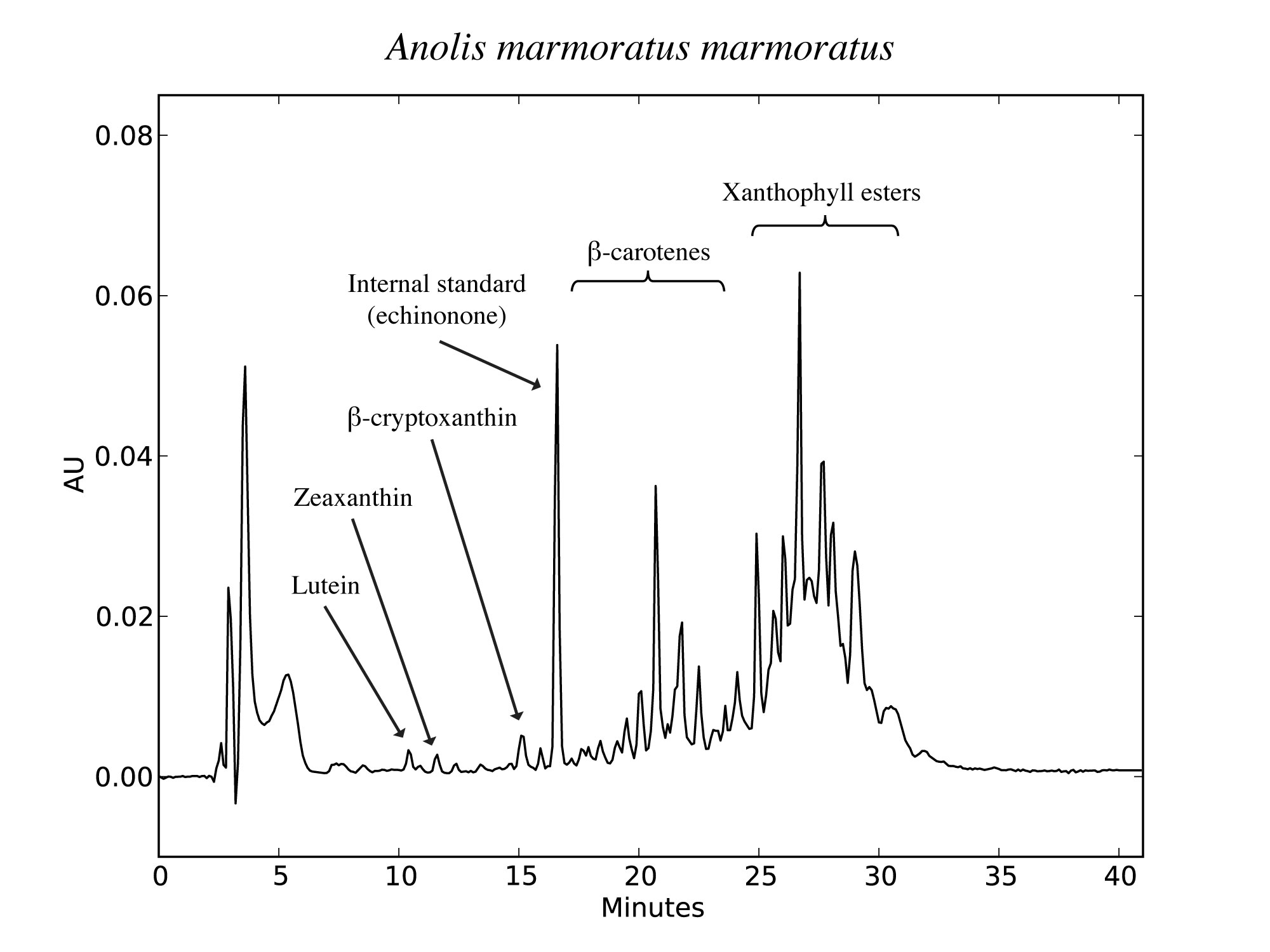


Table S5. The analysis of variance (ANOVA) reveals that all comparisons between subspecies and carotenoid pigments are significant (p < 0.05). The column names abbreviated as follows: degrees of freedom (df), sum of squares (sum sq), mean sum of squares (mean sq), F test statistic (F value), and p-value (Pr>(F)). The tukey HDS test identifies the only biologically relevant difference, a difference between the same pigment in the two subspecies, that is significant is the concentration of xanthophyll ester. The column names are comparison, the difference in the observed means (diff), and the p-value after adjustment for the multiple comparisons (p adj). Subspecies are abbreviated as follows: *A. m. marmoratus* (A.m.mar) and *A. m. speciosus* (A. m. spe).

**ANOVA:**

|  | Df | Sum Sq | Mean Sq | F value | Pr(>F) |
| --- | --- | --- | --- | --- | --- |
| Subspecies | 1 | 93906 | 93906 | 11.895 | 0.00134 |
| Carotenoid | 4 | 460436 | 115109 | 14.58 | 1.98E-07 |
| Subspecies:Carotenoid | 4 | 174352 | 43588 | 5.521 | 0.00123 |

**Tukey’s Honest Significant Difference (HSD) Test:**

| Subspecies vs pigment | diff | p adj |
| --- | --- | --- |
| A.m.spe:zeaxanthin-A.m.mar:xanthophyll ester | -397.06 | 0.0000006 |
| A.m.mar:xanthophyll ester-A.m.mar:lutein | 396.35 | 0.0000007 |
| A.m.mar:xanthophyll ester-A.m.spe:lutein | 395.95 | 0.0000007 |
| A.m.mar:zeaxanthin-A.m.mar:xanthophyll ester | -395.34 | 0.0000007 |
| A.m.mar:xanthophyll ester-A.m.mar:Β-cryptoxanthin | 388.62 | 0.000001 |
| A.m.mar:xanthophyll ester-A.m.spe:Β-cryptoxanthin | 385.90 | 0.0000012 |
| A.m.mar:xanthophyll ester-A.m.spe:carotene | 363.08 | 0.0000045 |
| A.m.spe:xanthophyll ester-A.m.mar:xanthophyll ester | -297.32 | 0.0001846 |
| A.m.mar:xanthophyll ester-A.m.mar:carotene | 225.62 | 0.0085512 |
| A.m.spe:zeaxanthin-A.m.mar:carotene | -171.44 | 0.1003879 |
| A.m.mar:lutein-A.m.mar:carotene | -170.73 | 0.103259 |
| A.m.spe:lutein-A.m.mar:carotene | -170.34 | 0.10489 |
| A.m.mar:zeaxanthin-A.m.mar:carotene | -169.72 | 0.1074471 |
| A.m.mar:carotene-A.m.mar:Β-cryptoxanthin | 163.00 | 0.1390731 |
| A.m.mar:carotene-A.m.spe:Β-cryptoxanthin | 160.28 | 0.1538735 |
| A.m.spe:carotene-A.m.mar:carotene | -137.46 | 0.3274115 |
| A.m.spe:zeaxanthin-A.m.spe:xanthophyll ester | -99.75 | 0.746167 |
| A.m.spe:xanthophyll ester-A.m.mar:lutein | 99.03 | 0.7535975 |
| A.m.spe:xanthophyll ester-A.m.spe:lutein | 98.64 | 0.7577055 |
| A.m.mar:zeaxanthin-A.m.spe:xanthophyll ester | -98.03 | 0.7639872 |
| A.m.spe:xanthophyll ester-A.m.mar:Β-cryptoxanthin | 91.31 | 0.8282031 |
| A.m.spe:xanthophyll ester-A.m.spe:Β-cryptoxanthin | 88.58 | 0.8514349 |
| A.m.spe:xanthophyll ester-A.m.mar:carotene | -71.70 | 0.9534047 |
| A.m.spe:xanthophyll ester-A.m.spe:carotene | 65.76 | 0.972826 |
| A.m.spe:zeaxanthin-A.m.spe:carotene | -33.98 | 0.9998067 |
| A.m.mar:lutein-A.m.spe:carotene | -33.27 | 0.9998376 |
| A.m.spe:lutein-A.m.spe:carotene | -32.87 | 0.9998529 |
| A.m.mar:zeaxanthin-A.m.spe:carotene | -32.26 | 0.9998741 |
| A.m.spe:carotene-A.m.mar:Β-cryptoxanthin | 25.54 | 0.9999825 |
| A.m.spe:carotene-A.m.spe:Β-cryptoxanthin | 22.82 | 0.9999934 |
| A.m.spe:Β-cryptoxanthin-A.m.mar:Β-cryptoxanthin | 2.73 | 1 |
| A.m.mar:lutein-A.m.mar:Β-cryptoxanthin | -7.73 | 1 |
| A.m.spe:lutein-A.m.mar:Β-cryptoxanthin | -7.33 | 1 |
| A.m.mar:zeaxanthin-A.m.mar:Β-cryptoxanthin | -6.72 | 1 |
| A.m.spe:zeaxanthin-A.m.mar:Β-cryptoxanthin | -8.44 | 1 |
| A.m.mar:lutein-A.m.spe:Β-cryptoxanthin | -10.46 | 1 |
| A.m.spe:lutein-A.m.spe:Β-cryptoxanthin | -10.06 | 1 |
| A.m.mar:zeaxanthin-A.m.spe:Β-cryptoxanthin | -9.45 | 1 |
| A.m.spe:zeaxanthin-A.m.spe:Β-cryptoxanthin | -11.17 | 1 |
| A.m.spe:lutein-A.m.mar:lutein | 0.40 | 1 |
| A.m.mar:zeaxanthin-A.m.mar:lutein | 1.01 | 1 |
| A.m.spe:zeaxanthin-A.m.mar:lutein | -0.71 | 1 |
| A.m.mar:zeaxanthin-A.m.spe:lutein | 0.61 | 1 |
| A.m.spe:zeaxanthin-A.m.spe:lutein | -1.11 | 1 |
| A.m.spe:zeaxanthin-A.m.mar:zeaxanthin | -1.72 | 1 |

Table S6. Sample information from twenty low-coverage Nextera libraries. Reads were mapped to the *Anolis carolinensis* reference genome (AnoCar2, ensembl version 67) with Stampy (version 1.0.18, r1526). Mapped and unmapped reads were calculated with ‘idxstats’ from Samtools (version 0.1.18, r982:295). Percent duplicates, which can be interpreted as the percent of PCR duplicated reads, were calculated with ‘MarkDuplicates’ with Picard command-line tools (version 1.72).

| Sample identifier | Mapped  reads | Unmapped  reads | Proportion  mapped | Percent  duplicates | Mean  coverage |
| --- | --- | --- | --- | --- | --- |
| CEJ010 | 31,000,195 | 2,161,695 | 0.93 | 0.067 | 1.5 |
| CEJ013 | 34,677,170 | 2,284,646 | 0.94 | 0.066 | 1.67 |
| CEJ019 | 32,932,812 | 2,230,908 | 0.94 | 0.064 | 1.64 |
| CEJ020 | 20,492,808 | 2,043,754 | 0.91 | 0.059 | 1.05 |
| CEJ035 | 31,081,113 | 2,260,251 | 0.93 | 0.064 | 1.52 |
| CEJ036 | 15,267,208 | 1,455,016 | 0.91 | 0.052 | 0.77 |
| CEJ037 | 12,489,406 | 1,359,970 | 0.90 | 0.052 | 0.64 |
| CEJ039 | 27,760,410 | 2,231,110 | 0.93 | 0.064 | 1.39 |
| CEJ040 | 40,353,416 | 2,365,148 | 0.94 | 0.086 | 1.71 |
| CEJ041 | 23,127,096 | 2,284,080 | 0.91 | 0.057 | 1.19 |
| CEJ082 | 30,109,949 | 2,261,709 | 0.93 | 0.061 | 1.5 |
| CEJ083 | 33,942,841 | 2,105,525 | 0.94 | 0.066 | 1.63 |
| CEJ084 | 44,943,034 | 2,417,372 | 0.95 | 0.085 | 1.88 |
| CEJ085 | 21,848,121 | 1,618,751 | 0.93 | 0.053 | 1.07 |
| CEJ088 | 45,561,414 | 2,535,924 | 0.95 | 0.087 | 1.92 |
| CEJ095 | 35,397,257 | 2,605,861 | 0.93 | 0.066 | 1.76 |
| CEJ097 | 19,037,667 | 2,010,709 | 0.90 | 0.055 | 0.98 |
| CEJ106 | 23,252,864 | 1,934,418 | 0.92 | 0.058 | 1.18 |
| CEJ107 | 32,947,009 | 1,828,645 | 0.95 | 0.071 | 1.5 |
| CEJ108 | 29,754,127 | 2,443,783 | 0.92 | 0.064 | 1.5 |

Table S7. Sample information from 48 RAD libraries. Reads were mapped to the *Anolis carolinensis* reference genome (AnoCar2, ensembl version 67) with bowtie2 (version 2.0.0-beta5). Mapped and unmapped reads were calculated with ‘idxstats’ from samtools (version 0.1.18, r982:295). Percent duplicates are not reported because many reads are expected to have identical starting positions because they originate at restriction sites.

| Sample identifier | Mapped  Reads | Unmapped Reads | Proportion Mapped |
| --- | --- | --- | --- |
| CEJ021 | 62,602 | 95,143 | 0.40 |
| CEJ035 | 155,129 | 205,498 | 0.43 |
| CEJ036 | 155,263 | 207,010 | 0.43 |
| CEJ037 | 196,385 | 265,597 | 0.43 |
| CEJ039 | 94,767 | 127,518 | 0.43 |
| CEJ040 | 208,392 | 243,587 | 0.46 |
| CEJ041 | 156,814 | 184,391 | 0.46 |
| CEJ082 | 30,313 | 45,422 | 0.40 |
| CEJ084 | 112,487 | 145,656 | 0.44 |
| CEJ085 | 70,570 | 84,013 | 0.46 |
| CEJ088 | 69,468 | 82,804 | 0.46 |
| CEJ100 | 90,243 | 108,132 | 0.45 |
| CEJ106 | 112,360 | 133,319 | 0.46 |
| CEJ107 | 193,252 | 222,936 | 0.46 |
| CEJ108 | 291,159 | 351,648 | 0.45 |
| CEJ110 | 205,051 | 214,181 | 0.49 |
| CEJ111 | 201,324 | 206,836 | 0.49 |
| CEJ112 | 157,378 | 162,679 | 0.49 |
| CEJ120 | 124,067 | 126,088 | 0.50 |
| CEJ121 | 207,164 | 210,820 | 0.50 |
| CEJ122 | 234,550 | 237,459 | 0.50 |
| CJS1974 | 303,235 | 309,754 | 0.49 |
| CJS1976 | 125,362 | 128,715 | 0.49 |
| CJS1978 | 238,126 | 334,249 | 0.42 |
| CJS1979 | 219,390 | 305,013 | 0.42 |
| CJS1980 | 148,747 | 209,353 | 0.42 |
| CJS1982 | 148,473 | 208,410 | 0.42 |
| CEJ092 | 155,225 | 177,874 | 0.47 |
| CEJ093 | 200,184 | 239,469 | 0.46 |
| CEJ094 | 64,473 | 77,144 | 0.46 |
| CEJ114 | 137,802 | 142,443 | 0.49 |
| CEJ115 | 119,568 | 120,420 | 0.50 |
| CEJ116 | 119,442 | 120,659 | 0.50 |
| CEJ117 | 228,906 | 234,710 | 0.49 |
| CJS2066 | 302,445 | 426,215 | 0.42 |
| CJS2067 | 306,768 | 436,911 | 0.41 |
| CJS2068 | 95,814 | 132,529 | 0.42 |
| CJS2069 | 284,076 | 395,033 | 0.42 |
| CJS2072 | 181,196 | 248,590 | 0.42 |
| CJS2073 | 284,844 | 404,467 | 0.41 |
| CJS2074 | 190,146 | 265,186 | 0.42 |
| CEJ028 | 92,970 | 127,791 | 0.42 |
| CEJ029 | 116,363 | 161,445 | 0.42 |
| CEJ030 | 74,445 | 101,161 | 0.42 |
| CEJ031 | 120,596 | 162,588 | 0.43 |
| CEJ032 | 111,483 | 149,886 | 0.43 |
| CEJ033 | 72,201 | 99,651 | 0.42 |
| CEJ034 | 102,276 | 137,600 | 0.43 |

Table S8. Genes that intersect at least one fixed SNP.

| Chrm | Start | Stop | Gene name | Fixed SNVs | SNVs/ kb |
| --- | --- | --- | --- | --- | --- |
| 1 | 119,835,491 | 119,838,333 | *gbx2* | 2 | 0.704 |
| 1 | 120,144,930 | 120,162,167 | *cxcr7* | 4 | 0.232 |
| 1 | 120,295,312 | 120,333,442 | *cops8* | 8 | 0.210 |
| 1 | 123,843,123 | 123,856,597 | ASU_ACAR_G.861 | 2 | 0.148 |
| 1 | 123,674,066 | 123,702,692 | ASU_ACAR_G.516 | 4 | 0.140 |
| 1 | 123,667,664 | 123,792,002 | *col6a3* | 17 | 0.137 |
| 1 | 119,992,527 | 120,054,757 | ASU_ACAR_G.1595 | 8 | 0.129 |
| 1 | 124,569,780 | 124,659,146 | *spred2* | 9 | 0.101 |
| 1 | 123,366,164 | 123,419,914 | *pcbp3* | 5 | 0.093 |
| 1 | 123,510,012 | 123,601,715 | *mlph* | 8 | 0.087 |
| 1 | 119,878,878 | 119,923,750 | *asb18* | 3 | 0.067 |
| 1 | 119,945,098 | 119,975,852 | ASU_ACAR_G.1896 | 2 | 0.065 |
| 1 | 123,480,220 | 123,496,446 | *rab17* | 1 | 0.062 |
| 1 | 120,646,948 | 120,860,206 | *wdpcp* | 7 | 0.033 |
| 1 | 123,059,468 | 123,091,621 | *lss* | 1 | 0.031 |
| 1 | 118,795,851 | 118,843,816 | *usp40* | 1 | 0.021 |
| 1 | 117,147,355 | 117,196,556 | *mreg* | 1 | 0.020 |
| 1 | 118,939,032 | 119,001,512 | *sh3bp4* | 1 | 0.016 |
| 1 | 119,299,304 | 119,366,110 | *trpm8* | 1 | 0.015 |
| 1 | 116,842,704 | 116,943,930 | *fn1* | 1 | 0.010 |
| 1 | 120,920,940 | 121,218,361 | *ehbp1* | 2 | 0.007 |
| 1 | 117,398,453 | 117,547,709 | *lrrfip1* | 1 | 0.007 |
| 1 | 119,494,704 | 119,801,005 | ASU_ACAR_G.1239 | 2 | 0.007 |
| 1 | 118,407,016 | 118,568,515 | *hdac4* | 1 | 0.006 |
| 1 | 115,654,185 | 116,352,180 | *spag16* | 4 | 0.006 |
| 2 | 98,508,848 | 98,515,985 | ASU_ACAR_G.4239 | 1 | 0.140 |
| 2 | 98,774,658 | 98,803,896 | ASU_ACAR_G.2804 | 4 | 0.137 |
| 2 | 98,479,182 | 98,489,550 | *ccdc137* | 1 | 0.096 |
| 2 | 98,286,852 | 98,348,971 | *kif19* | 5 | 0.080 |
| 2 | 98,411,171 | 98,445,852 | *gprc5c* | 2 | 0.058 |
| 2 | 98,089,216 | 98,106,933 | *rpl38* | 1 | 0.056 |
| 2 | 98,489,332 | 98,508,518 | *c17orf90* | 1 | 0.052 |
| 2 | 98,938,873 | 98,965,443 | ASU_ACAR_G.3616 | 1 | 0.038 |
| 2 | 99,066,280 | 99,174,628 | *bahcc1* | 4 | 0.037 |
| 2 | 98,117,434 | 98,243,345 | *ttyh2* | 3 | 0.024 |
| 2 | 99,204,345 | 99,313,730 | *slc38a10* | 2 | 0.018 |
| 3 | 49,341,212 | 49,372,986 | ASU_ACAR_G.6170 | 5 | 0.157 |
| 3 | 49,373,287 | 49,386,266 | ASU_ACAR_G.5477 | 1 | 0.077 |
| 3 | 49,280,721 | 49,305,751 | ASU_ACAR_G.5128 | 1 | 0.040 |
| 3 | 49,386,391 | 49,454,228 | ASU_ACAR_G.5200 | 2 | 0.029 |
| 3 | 53,442,663 | 53,571,381 | *nps* | 1 | 0.008 |
| 5 | 91,047,732 | 91,061,160 | ASU_ACAR_G.8643 | 4 | 0.298 |
| 5 | 91,349,266 | 91,370,664 | *arntl2* | 5 | 0.234 |
| 5 | 94,759,018 | 94,813,911 | *phtf2* | 9 | 0.164 |
| 5 | 94,734,446 | 94,753,219 | *phtf2* | 3 | 0.160 |
| 5 | 90,723,760 | 90,737,227 | *sspn* | 2 | 0.149 |
| 5 | 94,301,911 | 94,387,054 | ASU_ACAR_G.9356 | 12 | 0.141 |
| 5 | 91,101,471 | 91,139,803 | *tm7sf3* | 5 | 0.130 |
| 5 | 90,033,023 | 90,056,227 | *sephs1* | 3 | 0.129 |
| 5 | 92,565,814 | 92,574,008 | ASU_ACAR_G.9238 | 1 | 0.122 |
| 5 | 90,759,970 | 91,032,630 | ASU_ACAR_G.9639 | 31 | 0.114 |
| 5 | 92,483,966 | 92,565,424 | ASU_ACAR_G.8729 | 9 | 0.110 |
| 5 | 92,481,254 | 92,547,892 | *hgf* | 7 | 0.105 |
| 5 | 93,356,786 | 93,387,109 | *gnai1* | 3 | 0.099 |
| 5 | 91,064,533 | 91,085,807 | *asun* | 2 | 0.094 |
| 5 | 90,076,684 | 90,152,191 | *bend7* | 7 | 0.093 |
| 5 | 91,391,137 | 91,542,974 | *ppfibp1* | 14 | 0.092 |
| 5 | 93,761,912 | 94,694,509 | *magi2* | 77 | 0.083 |
| 5 | 94,882,679 | 94,967,846 | ASU_ACAR_G.9343 | 7 | 0.082 |
| 5 | 95,018,720 | 95,087,720 | *pion* | 5 | 0.072 |
| 5 | 91,261,050 | 91,318,784 | *stk38l* | 4 | 0.069 |
| 5 | 95,098,726 | 95,161,797 | *ccdc146* | 4 | 0.063 |
| 5 | 91,546,567 | 91,578,856 | *cyb5r3* | 2 | 0.062 |
| 5 | 89,911,937 | 89,944,937 | *mcm10* | 2 | 0.061 |
| 5 | 91,210,091 | 91,227,438 | ASU_ACAR_G.9116 | 1 | 0.058 |
| 5 | 96,404,072 | 96,484,222 | *mll5* | 4 | 0.050 |
| 5 | 91,802,251 | 92,347,805 | ASU_ACAR_G.8645 | 27 | 0.049 |
| 5 | 96,338,532 | 96,402,302 | ASU_ACAR_G.9435 | 3 | 0.047 |
| 5 | 96,911,503 | 96,932,942 | ASU_ACAR_G.9674 | 1 | 0.047 |
| 5 | 93,086,140 | 93,209,248 | *cd36* | 5 | 0.041 |
| 5 | 92,782,560 | 93,057,082 | *sema3c* | 11 | 0.040 |
| 5 | 89,976,817 | 90,029,508 | *phyh* | 2 | 0.038 |
| 5 | 91,143,278 | 91,170,041 | *med21* | 1 | 0.037 |
| 5 | 96,070,870 | 96,361,913 | *lhfpl3* | 9 | 0.031 |
| 5 | 92,422,593 | 92,459,019 | ASU_ACAR_G.9652 | 1 | 0.027 |
| 5 | 93,222,769 | 93,295,736 | *gnat3* | 2 | 0.027 |
| 5 | 92,609,151 | 92,719,185 | ASU_ACAR_G.9577 | 3 | 0.027 |
| 5 | 94,842,790 | 94,881,580 | *rsbn1l* | 1 | 0.026 |
| 5 | 91,145,587 | 91,185,218 | ASU_ACAR_G.9036 | 1 | 0.025 |
| 5 | 95,484,817 | 95,699,674 | *reln* | 4 | 0.019 |
| 5 | 96,483,250 | 96,616,399 | *srpk2* | 2 | 0.015 |
| 5 | 90,177,605 | 90,467,331 | ASU_ACAR_G.9392 | 3 | 0.010 |
| 5 | 97,160,785 | 97,259,958 | *prkar2b* | 1 | 0.010 |
| 6 | 58,874,533 | 58,911,992 | *hgsnat* | 4 | 0.107 |
| 6 | 59,047,475 | 59,095,011 | *uso1* | 5 | 0.105 |
| 6 | 58,911,486 | 58,940,401 | *ints10* | 3 | 0.104 |
| 6 | 59,363,344 | 59,380,599 | *fabp1* | 1 | 0.058 |
| 6 | 58,293,349 | 58,385,173 | ASU_ACAR_G.10108 | 5 | 0.054 |
| 6 | 58,753,098 | 58,847,042 | *slc20a2* | 4 | 0.043 |
| 6 | 58,235,094 | 58,287,278 | *uba6* | 2 | 0.038 |
| 6 | 59,145,786 | 59,171,970 | *cdkl2* | 1 | 0.038 |
| 6 | 58,395,777 | 58,471,894 | ASU_ACAR_G.10619 | 2 | 0.026 |
| 6 | 58,520,646 | 58,563,241 | *ythdc1* | 1 | 0.023 |
| 6 | 61,824,974 | 61,884,161 | *ccr10* | 1 | 0.017 |
| 6 | 57,422,324 | 57,490,624 | *tmem245* | 1 | 0.015 |
| 6 | 62,189,732 | 62,264,752 | *wnk4* | 1 | 0.013 |
| 6 | 57,222,624 | 57,383,851 | ASU_ACAR_G.9876 | 1 | 0.006 |
